# *Leptospira interrogans* serovar Copenhageni Harbors Two *lexA* Genes Involved in SOS Response

**DOI:** 10.1371/journal.pone.0076419

**Published:** 2013-10-03

**Authors:** Luciane S. Fonseca, Josefa B. da Silva, Juliana S. Milanez, Claudia B. Monteiro-Vitorello, Leonardo Momo, Zenaide M. de Morais, Silvio A. Vasconcellos, Marilis V. Marques, Paulo L. Ho, Renata M. A. da Costa

**Affiliations:** 1 Centro de Biotecnologia, Instituto Butantan, São Paulo, Brazil; 2 Departamento de Bioquímica, Instituto de Química, Universidade de São Paulo, São Paulo, Brazil; 3 Departamento de Genética, Escola Superior de Agricultura Luiz de Queiroz, Piracicaba, Brazil; 4 Centro de Ciências Naturais e Humanas, Universidade Federal do ABC, Santo André, Brazil; 5 Departamento de Medicina Veterinária Preventiva e Saúde Animal, Universidade de São Paulo, Brazil; 6 Departamento de Microbiologia, Instituto de Ciências Biomédicas, Universidade de São Paulo, Brazil; Florida International University, United States of America

## Abstract

Bacteria activate a regulatory network in response to the challenges imposed by DNA damage to genetic material, known as the SOS response. This system is regulated by the RecA recombinase and by the transcriptional repressor lexA. *Leptospira interrogans* is a pathogen capable of surviving in the environment for weeks, being exposed to a great variety of stress agents and yet retaining its ability to infect the host. This study aims to investigate the behavior of *L. interrogans* serovar Copenhageni after the stress induced by DNA damage. We show that *L. interrogans* serovar Copenhageni genome contains two genes encoding putative LexA proteins (*lexA1* and *lexA2*) one of them being potentially acquired by lateral gene transfer. Both genes are induced after DNA damage, but the steady state levels of both LexA proteins drop, probably due to auto-proteolytic activity triggered in this condition. In addition, seven other genes were up-regulated following UV-C irradiation, *recA*, *recN, dinP*, and four genes encoding hypothetical proteins. This set of genes is potentially regulated by LexA1, as it showed binding to their promoter regions. All these regions contain degenerated sequences in relation to the previously described SOS box, TTTGN _5_CAAA. On the other hand, LexA2 was able to bind to the palindrome TTGTAN_10_TACAA, found in its own promoter region, but not in the others. Therefore, the *L. interrogans* serovar Copenhageni SOS regulon may be even more complex, as a result of LexA1 and LexA2 binding to divergent motifs. New possibilities for DNA damage response in *Leptospira* are expected, with potential influence in other biological responses such as virulence.

## Introduction

*Leptospira interrogans* is one of the etiologic agents of leptospirosis, a worldwide disease with important economic and public health consequences, in particular to developing tropical countries [1,2]. The leptospires can infect a wide range of mammalian species that compose their natural reservoir, colonizing the kidneys, and being shed in the urine during the whole life of these animals [3]. There are nine pathogenic species of *Leptospira*, divided in more than 260 serovars [4]. In Brazil, the majority of the leptospirosis cases in humans is the result of infection with serovar Copenhageni [1]. In spite of its social and economic impact, the molecular mechanisms of *Leptospira* pathogenesis are still poorly understood, as a consequence of the difficulties in their genetic manipulation. Particularly, *L. interrogans* serovar Copenhageni remains one of the serovars most refractory to genetic transformation and only two mutants were so far obtained by targeted mutagenesis [5,6].

*L. interrogans* can survive in water or mud for weeks, after which they are still able to infect the host. These leptospires are exposed to a wide spectrum of DNA-damaging agents, from sun radiation and heavy metals to oxidative stress and antibiotics [7,8]. One of the most important mechanisms employed by bacteria to deal with stress induced by DNA damage is the SOS response. This regulatory network controls DNA repair, error prone DNA replication, cell division and mobilization of phages and transposable elements in *E. coli* [9-12]. The expression of these genes is repressed by LexA, which dimerizes and binds to operators in their promoters at regions called SOS boxes [13,14]. The induction of the SOS response is triggered by genomic structure alterations that generate single-stranded DNA (ssDNA), which is sensed by the cells during replication. RecA recognizes and interacts with these damaged regions, acquiring an active conformation and playing a role as a co-factor in the self-cleavage reaction of LexA. The cleavage generally occurs in a peptide bond flanked by Ala-Gly residues near the center of the protein sequence, disrupting LexA dimerization, which in turn, reduces DNA binding and allows transcription initiation [15-18]. Once the damage is repaired, the level of activated RecA drops, and newly synthesized LexA (whose expression is usually under its own regulation) binds to SOS boxes again, returning the system to the non-induced state [11].

The *lexA* gene is found in most bacterial taxa, with few exceptions. However, the set of genes directly repressed by LexA diverges substantially. This is in part consequence of the great degree of sequence heterogeneity among SOS boxes. The sequence variation is a result of the low conservation in amino acid residues in the DNA binding domain of LexA among different groups of bacteria [19]. Although the majority of bacteria has only one copy of *lexA*, genomic sequences of some organisms underwent duplication or lateral gene transfer, resulting in two genes coding LexA proteins regulating different sets of genes [20-22]. As a consequence, the SOS response is a very unique and complex regulatory network, with a remarkable flexibility of LexA-regulated genes.

The SOS response has important consequences for bacterial physiology and for virulence mechanisms in pathogenic organisms [23,24]. All leptospiras sequenced to date harbor one copy of the *lexA* gene. Previous work reported that the LexA protein from *L. interrogans* serovar Lai (LA1447) [25] has activity of a transcriptional repressor, acting only on *recA* gene expression. Moreover, the identified SOS box palindrome ( **TTTG**CTATA**CAAA** ) was found only upstream of the *recA* gene. As such, *L. interrogans*, along with *Thermotoga maritima*, would be among the rare organisms in which LexA does not regulate its own transcription [26]

In this study we show that the DNA damage induced by UV-C irradiation triggered the SOS response in *L. interrogans* serovar Copenhageni. Analyzing the bacterium genome, we found a second *lexA* gene (*lexA2*) within a prophage-like region rich in genes encoding hypothetical proteins. Following the stress induced by UV-C irradiation, *L. interrogans* displayed filamentation and both LexA repressors were depleted, presumably as a consequence of self-cleavage. The expression levels of both *lexA* genes, as well as those of other seven genes, were increased from eight up to 12 hours after the UV-C treatment. LexA1 was able to bind to the promoter sequences of *recA* and *recN*, and competition assays indicate its binding to the promoters of the remaining UV-C induced transcripts, including the one containing *lexA1*. Not all the genes showing UV-C induction do have the exact previously described SOS box in their promoter regions, but alignment of these sequences showed the presence of imperfect palindromic dyads that could be LexA1 binding sites. On the other hand, LexA2 showed specific binding only to a sequence upstream of its own transcriptional unit. Therefore, the physiological response of *L. interrogans* serovar Copenhageni may be even more complex than in other bacteria, as LexA1 appears to have some flexibility to recognize degenerated SOS sequences.

## Material and Methods

### In silico *analysis*

All sequences used were obtained from Kyoto Encyclopedia of Genes and Genomes (KEGG) and National Center for Biotechnological Information (NCBI) databases. The secondary structure predictions were made by PsiPred [27], and search for structural domains in hypothetical protein sequences by HHPred (http://hhpred.tuebingen.mpg.de). Finally, the search for putative SOS box motifs was carried out by using the tool “genome scale DNA-pattern”, available from RSAT (http://rsat.ccb.sickkids.ca). The output returned all palindromes present in the upstream region of the genes (nucleotides +20 to -250 from the start codon).

### Phylogenetic analysis

A total of 48 protein sequences (Table S1) were obtained through BLAST searches using either LexA1 or LexA2 as query. MUSCLE [28,29] alignments were used to infer phylogenetic trees, constructed using maximum likelihood analysis with WAG substitution model in PhyML [30]. The robustness of the trees was assessed by aLRT [31]. Node support was assessed as the posterior probability from two independent runs, with four chains of 200,000 generations each (sampled at intervals of 100 generations with a burn-in of 1000 trees).

### Bacterial strains and growth conditions

*L. interrogans* serovar Copenhageni Strain FioCruz L1-130 and other serovars (Australis, Autumnalis, Bataviae, Canicola, Pomona, Pyrogenes, Hardjo) and *L. borgpetersenii* serovar Hardjobovis were obtained from Faculdade de Medicina Veterinária e Zootecnia (Universidade de São Paulo, Brazil), while the genomic DNA from serovars Smithi and Naam were obtained as described by da Silva et al. [32]. The growth and virulence maintenance were carried out according to da Silva et al. [33]. *E. coli* DH5α and BL21(DE3) Star pLysS were used for cloning and expression procedures, respectively. *E. coli* cells were grown at 37°C in LB medium containing the appropriate antibiotics.

### UV-C irradiation, survival curves and visualization

Virulent *L. interrogans* serovar Copenhageni L1-130 was cultivated until density of approximately 4x10^8^ cells/ml. Bacteria were transferred to 140mm diameter Petri dishes, conserving a thin layer of culture, and exposed for increasing times to a germicidal lamp (254 nm, rate 1 J.m^-^²^.^ s^-1^). After treatment, the same volume of fresh medium was added to stimulate cellular division and the culture was incubated at 30°C in the dark. Surviving bacteria were counted 24 hours post-treatment using a Petroff-Hausser counting chamber and survival frequency was calculated as the ratio of irradiated to non-irradiated cells. Cells were visualized by fluorescence microscopy after labeling with DAPI (4’,6-diamidino-2-phenylindole) [34] and measured using ImageJ software (http://rsbweb.nih.gov/ij). For RNA extraction, cells were collected 4, 8, 12 and 28h following UV-C exposure, as well as their non-treated counterparts, immediately frozen in liquid nitrogen and stored at -80°C until use.

### Recombinant protein expression and purification

All enzymes cited in this section were obtained from Fermentas (USA), and used according to the manufacturer instructions. The coding regions of *lexA1* (LIC12305) and *lexA2* (LIC12654) were codon-optimized for expression in *E. coli* (Genscript, USA). These sequences were cloned in the expression vector pAE [35] and the recombinant proteins were over-expressed in *E. coli* BL21(DE3) Star pLysS as described elsewhere [36]. The cells were harvested by centrifugation, ressuspended in 50 mM Tris-HCl (pH 6.3), 150 mM NaCl and lysed in a French press (Thermo Spectronic). The supernatant was applied to a 1 cm-diameter column containing 3 ml Ni^2+^-charged chelating Sepharose (GE Healthcare Life Sciences, USA). The proteins were eluted with 400 mM imidazole, which was removed by dialysis. Purified proteins were visualized by Coomassie blue staining after separation by 15% SDS-PAGE.

### Western blot

For immunoblotting, 20 ng of purified proteins and 40 µg or 150 µg of leptospiral extracts were separated by 15% SDS-PAGE and transferred to Hybond-P Polyvinylidene Difluoride (GE Healthcare Life Sciences, USA) membranes. Incubations and detection were carried out as described elsewhere [36], using anti-LexA1 in 1:5000 dilution, anti-LexA2 in 1:1000 and anti-LipL32 [37] in 1:5000. Anti-LexA2 serum was incubated with 200ng/µl purified LexA1 for 2h prior to use, to decrease cross-reactivity.

### DNA purification and PCR

Genomic DNA (gDNA) of *Leptospira* was isolated using DNAzol (Invitrogen), following the manufacturer instructions, and quantified by NanoDrop (Thermo Scientific, USA) spectrophotometer. It was used as positive control in RT-PCR experiments (see below).

### RNA manipulation and quantitative PCR

Total RNA was prepared using Trizol (Invitrogen, USA) according to manufacturer instructions and treated with DNaseI (Fermentas, USA) to avoid gDNA contamination. Purified RNA was quantified by NanoDrop. Next, 1 µg of RNA was used as template for the complementary DNA (cDNA) synthesis by the reverse transcriptase M-MuLV (New England Biolabs, USA), using random hexamers. Reverse transcriptase-PCR (RT-PCR) to assess the transcription organization of *lexA1* and *lexA2* vicinities were carried out using 1 µl of 1:5 cDNA as template, for 40 cycles. Quantitative PCR (qPCR) was performed with SYBR Green Master Mix (Applied Biosystems, USA), using 1 µl of 1:100 cDNA in 12 µl reactions. Reactions were set at the default profile of Applied Biosystems 7300 Real-Time PCR System (2min at 50°C and 10min at 95°C, followed by 40 cycles of 15s at 90°C and 1min at 60°C). A posterior dissociation cycle (15s at 96°C, 20s at 60°C, 15s at 90°C and 15s at 60°C) was added to discard the existence of any contaminating product. Fold change was calculated by the 2^-∆∆Ct^ method, using the 16S as internal control. Each experiment was repeated three times, with biological replica. Data were analyzed through GraphPad Prism5, where the variance was assessed by one way ANOVA and significance of differences by Dunnett post-test. Oligonucleotides used in these experiments are compiled in Table S2.

### Electrophoretic Mobility Shift Assay (EMSA)

EMSA was performed using DIG Gel Shift Kit (Roche), following the manufacturer instructions. Probes amplified by PCR (Table S2) were purified by GFX™ PCR DNA and Gel Band Purification Kit (GE Healthcare, USA), quantified by NanoDrop and labeled with a terminal DIG. Alternatively, probes were labeled with [γ ^32^P ATP] by T4 Polynucleotide Kinase (Fermentas) and column purified (GenElute PCR Clean-Up Kit, Qiagen). Binding reactions were carried out in ice, in binding buffer provided in the kit, using poly[d(A-T)] as unspecific competitor. Solutions containing 1.55 fmol of labeled probes were incubated with 40 µg leptospiral extracts or 80 ng purified LexA2 for 20min. In antibody blockade assay, extracts were incubated with 1 µl of anti-LexA1 or preimmune sera for 30min in ice prior to the addition of the probe. In competition assays, non-labeled probes were added to the binding reaction after the labeled one, in 200 fold excess (310 fmol). Mixtures were loaded onto a 5% non-denaturing 0.5x TBE gel pre-run at 80 V for 90min. DNA-protein complexes were separated at 80 V for 150min at 4°C and transferred to a Hybond-N (GE Healthcare, USA) nylon membrane using a Trans-Blot Semi-Dry Electrophoretic Transfer Cell (Bio-Rad, Germany) in TBE 0.5x for 90min at 5 V. Detection followed the manufacturer instructions, and the membranes were exposed to photographic films (Hyperfilm, ECL GE Healthcare, USA).

## Results

### *L. interrogans* *serovar* Copenhageni genome harbors a second *lexA* gene

**Figure 1:**
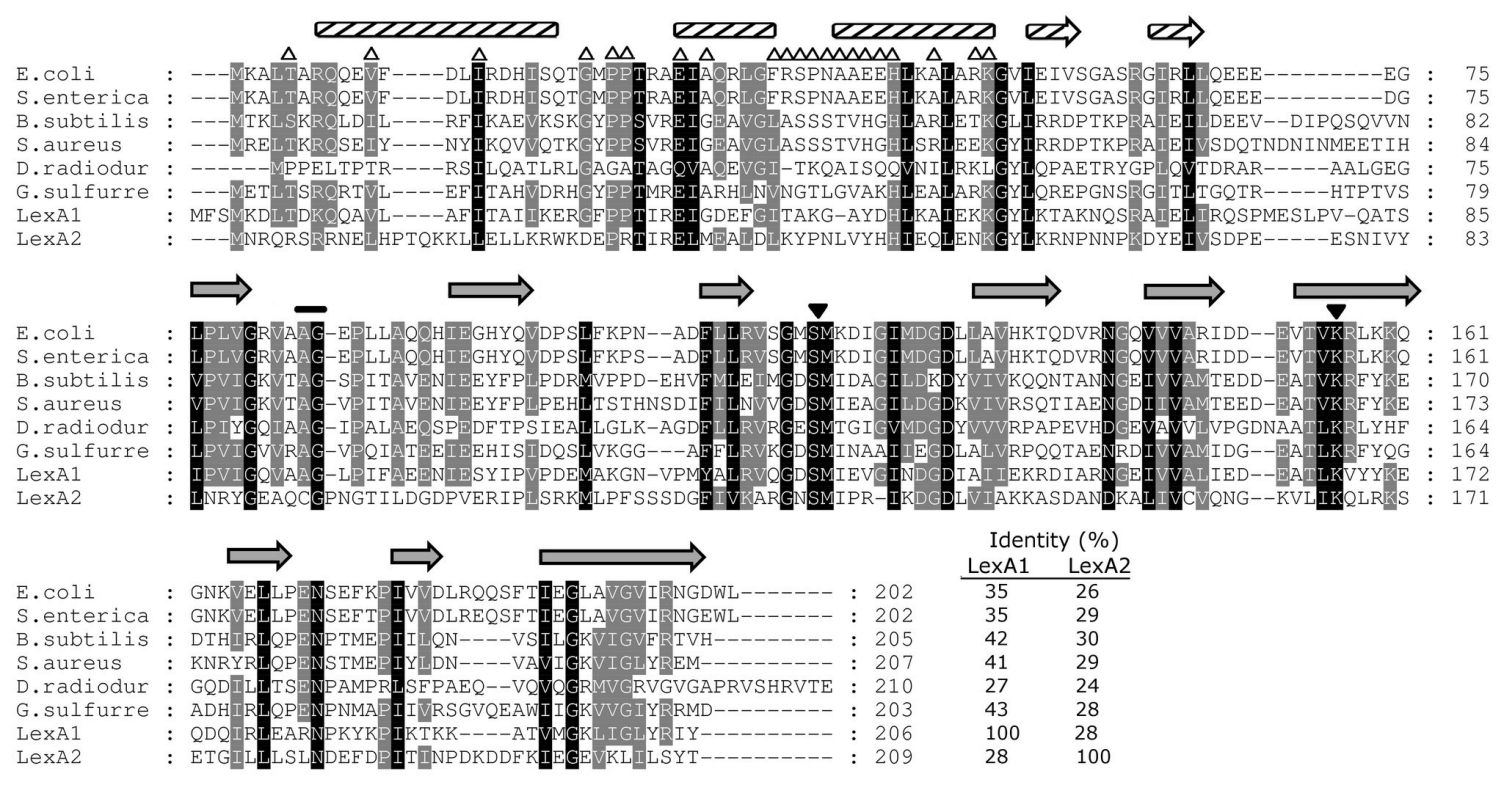
Comparison of LexA amino acid sequences. Amino acid sequence alignment and secondary structure prediction were carried out using *E. coli* LexA as reference. The amino-terminal region is composed of three helixes (striped rectangles) and β strands 1 and 2 (striped arrows), while the carboxy-terminal is composed of nine β strands (grey arrows). Arrowheads indicate the catalytic residues, and the bar indicates the residues flanking the scissile peptide bond. Open triangles represent residues that interact with DNA. The percentage of identity of each sequence to either *L. interrogans* serovar Copenhageni LexA proteins is indicated.

Analysis of the genome of *L. interrogans* serovar Copenhageni [38] revealed the presence of a second homologous gene for *lexA*, LIC12654. For clarity, we named it *lexA2*, whereas the gene identical to *lexA* from *L. interrogans* serovar Lai [25] was named *lexA1* (LIC12305). The LexA2 predicted amino acid sequence exhibits very low similarity with the known LexA proteins, sharing 28% of amino acid identity to LexA1. Nevertheless, the predicted secondary structure shows both DNA-binding and serine-protease domains compatible with LexA-like protein structure [16,39] (Figure 1). The catalytic residues Ser and Lys (indicated with arrowheads in Figure 1), located at the carboxy-terminal domain and typically 37 amino acids apart, are at positions 130 and 166 in LexA2, respectively, while the scissile peptide bond is probably located in Cys_92_-Gly_93_ (indicated with a bar in Figure 1). The helix-turn-helix structure of the amino-terminal domain of LexA2 is conserved. However, from the 21 amino acid residues potentially involved in DNA binding in this domain (labeled with Δ in Figure 1), 16 are different between LexA1 and LexA2. Since this is the domain responsible for the SOS box recognition, it is conceivable that both proteins must regulate different sets of genes.

### LexA2 coding sequence was possibly acquired through lateral gene transfer

**Figure 2:**
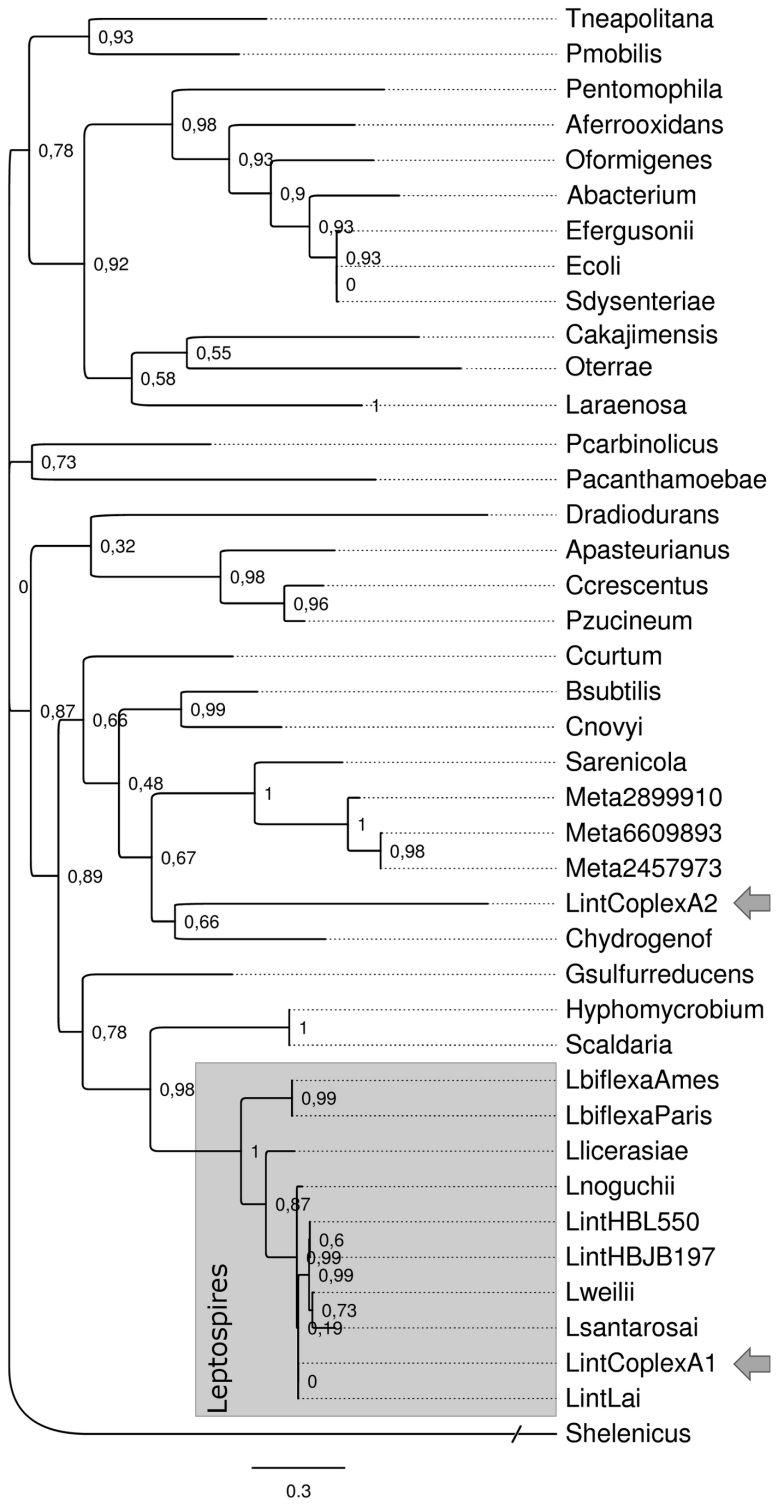
Phylogenetic analysis of LexA. Phylogenetic analysis was performed using LexA amino acid sequences from several bacteria. The leptospiras clade is highlighted by a grey box, and the sequences of the LexA proteins present in *L. interrogans* serovar Copenhageni are indicated with arrows. Local bootstrap values are attached to the internal nodes. Species code description and sequences used are compiled in Table S1.

The remarkable differences in amino acid sequence of LexA2 raised the question if it was acquired through lateral gene transfer. This hypothesis was tested through phylogenetic analyses (Figure 2). The multiple alignments were used to construct phylogenetic trees with maximum-likelihood algorithm. The distribution of the evolutionary distances and the tree topology reveals a long phylogenetic distance among the two *L. interrogans* serovar Copenhageni LexA repressors. The LexA2 protein grouped in a distinct clade with sequences from marine metagenomes, while LexA1 clustered with orthologous from other leptospires. Therefore, both coding sequences did not evolve together.

### Comparative genomic organization of genes *lexA1 and lexA2* vicinities

**Figure 3:**
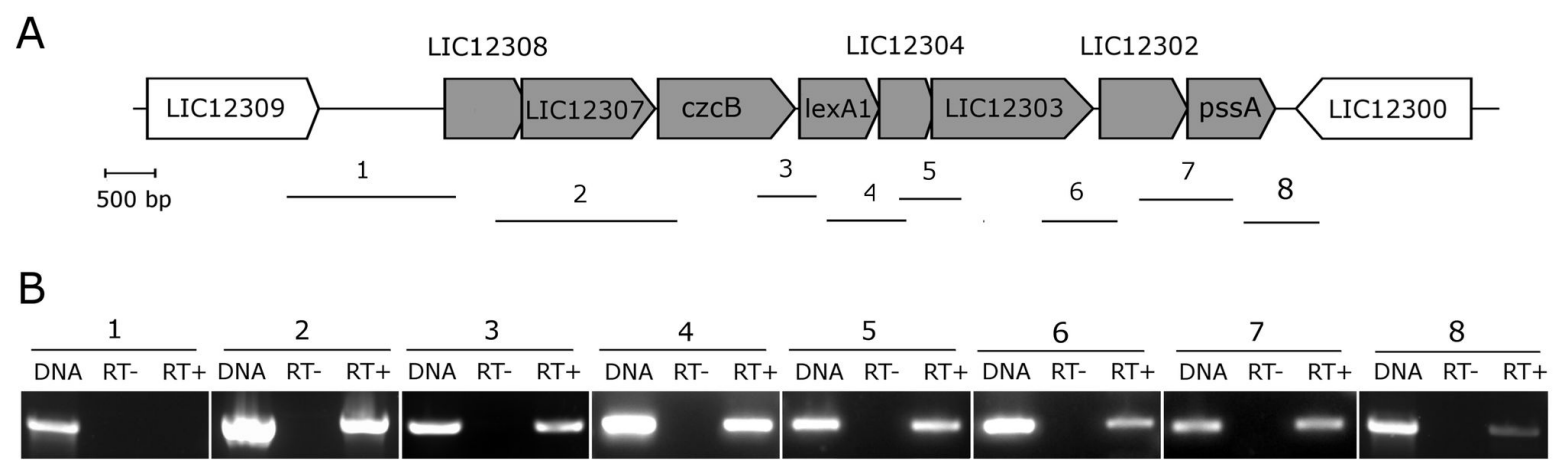
Genomic and transcriptional organization of the *lexA1* region. (A) Schematic representation of the *lexA1* genomic region. The arrows indicate the direction of transcription. The fragments amplified by the primer pairs used for the RT-PCR analysis are indicated by numbered lines below the genes. (B) Composite image of agarose gels from resulting RT-PCR reactions, using either genomic DNA (DNA), RNA (RT-) or cDNA (RT+) as templates. The numbers refer to the respective fragments shown in (A).

**Figure 4:**
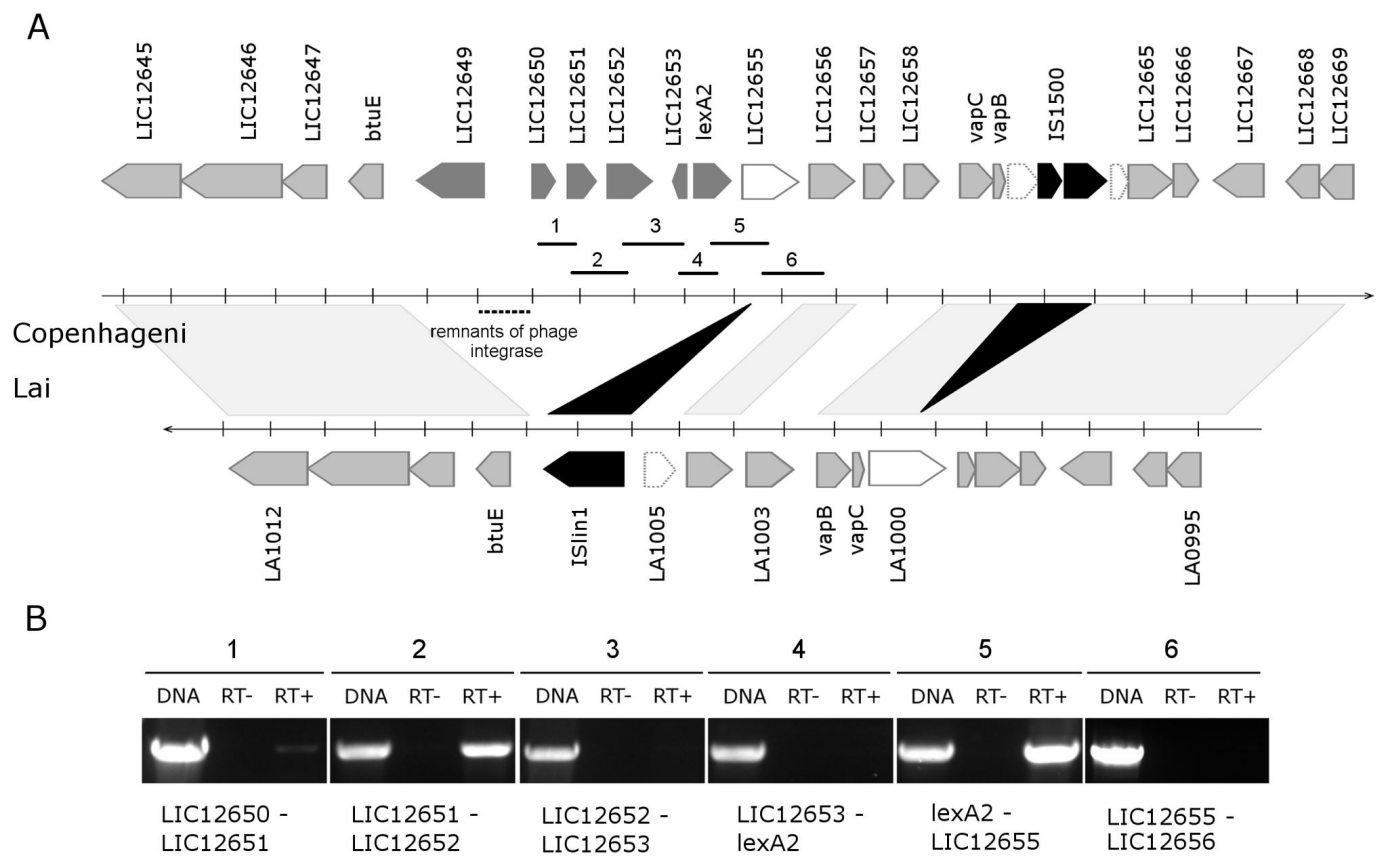
Genomic and transcriptional organization of the *lexA2* region. (A) Schematic representation of the *lexA2* genomic region from *L. interrogans* serovar Copenhageni (upper) compared to the equivalent region of serovar Lai (lower). Arrows represent predicted genes and transcription orientation. Light grey arrows represent genes orthologous between genomes, dark grey genes that are specific to Copenhageni and black arrows indicate genes encoding transposases. The white arrows represent genes with truncated versions in Lai genome (traced arrows) by insertion of IS elements. Remnants of a phage integrase are indicated by a traced line. The numbered bars below the genes indicate the amplified fragments corresponding to the primer pairs used in the RT-PCR analyses. (B) RT-PCR reactions, using either genomic DNA (gDNA), RNA (RT-) or cDNA (RT+) as templates, and primers flanking intergenic regions. The numbers refer to the respective fragments shown in (A).

The *lexA*1 and *lexA*2-containing regions are located within the large inversion which differentiates serovars Lai and Copenhageni genomes [40]. The *lexA1* gene vicinities of both genomes are identical, sharing 99% of nucleotide identity. This region is enriched with genes encoding peptidases and stress response proteins (Figure 3A). Besides the S24 peptidase *lexA1*, LIC12303 is annotated as a S41 peptidase and LIC12302 as a M22 peptidase. The hypothetical protein LIC12304 shows a structure similar to the xenobiotic response element (XRE) family of transcriptional regulators; *czcB* is a heavy metal efflux pump; and LIC12307 is a TolC superfamily transporter protein. The genomic organization, exhibiting less than 43 bp intergenic spaces, indicates a structure of an operon (Figure 3A) [41]. To investigate this hypothesis, primers were designed to amplify across intergenic regions of *L. interrogans* serovar Copenhageni cDNA. The result showed amplicons for all gene pairs, from LIC12308 to *pssA* (Figure 3B), suggesting the occurrence of an mRNA spanning this entire region. The *lexA*2 gene lies within a prophage-like region rich in genes encoding hypothetical proteins. The genome context of *lexA*2 resembles the remnant of an ancient phage infection, which has been subject of mutational decay and rearrangements leading to losses of most of the prophage genes. This mosaic architecture also harbors insertion sequence (IS) elements interrupting genes as compared to the equivalent region of serovar Lai (Figure 4A). BLASTX searches revealed the presence of remnants of a phage-integrase gene (Figure 4A) but no duplication or any potential site of insertion could be detected. RT-PCR analysis showed that all the genes surrounding *lexA*2 are probably expressed in normal culture conditions as three different transcripts: LIC12650-12652, LIC12653 and lexA2-LIC12655 (Figure 4B). While LIC12650 and LIC12651 encode phage-related genes, LIC12655 encodes a peptidase of the M28 family. The peculiar nature of this region suggests that both serovars Copenhageni and Lai at some point harbored these similar regions that have evolved apart mostly because of multiple events of insertions and rearrangements. We also investigated the presence of *lexA2* region in the genomes of other serovars and species of *Leptospira* that have been sequenced. MEGABLAST searches at the Whole-genome shotgun contigs database (wgs) at GenBank (Figure S1), together with tentative PCR amplification of *lexA2* using non-sequenced serovars DNA (Figure S2), revealed that this region is highly specific to Copenhageni.

### *Leptospira displays* filamentation after UV-C irradiation

**Figure 5:**
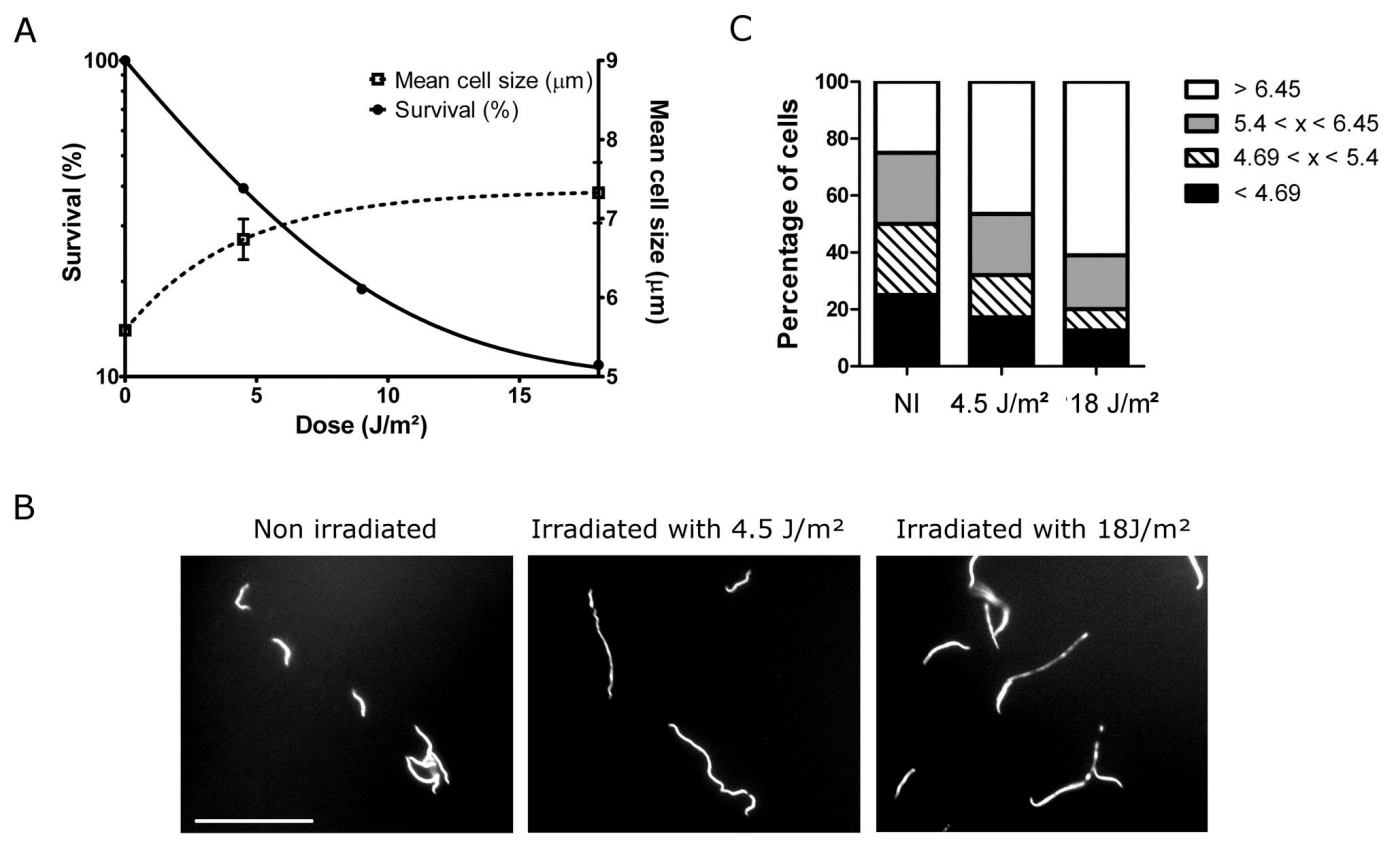
Phenotypic analyses of *L. interrogans* serovar Copenhageni after UV-C irradiation. (A) Relationship between survival after treatment with increasing doses of UV-C (left axis) and the mean size of the persistent cells (right axis). Cultures of bacteria were treated with increasing doses of UV-C, diluted in fresh medium and incubated during 24h in the dark. Surviving bacteria were counted and the frequency was calculated as the ratio of irradiated to non-irradiated cells. (B) Fluorescent microscopy of leptospires in cultures treated or not with UV-C, after staining with DAPI (1000x magnification). The bar represents 20 µm. (C) Frequencies of the size categories from treated and non-treated samples. Categories were determined by the size values (expressed in µm) that divided the non-irradiated culture in its four quartiles. All the data represent the average of three independent experiments.

To assess how *L. interrogans* serovar Copenhageni behaves after the stress induced by DNA damage, cells were exposed to increasing UV-C doses. A dose of 4.5 J.m^-2^ was sufficient to kill 50% of the cells, whereas after 18 J.m^-2^, only 10% of the cells survived (Figure 5A). Additionally, persistent UV-C-irradiated cells exhibited filamentation when compared to non-irradiated ones. Cultures were stained with DAPI after 24h of incubation and visualized by fluorescence microscopy. Since the genomic DNA in spirochetes occupies the whole intracellular space [34], the cells were homogeneously stained throughout their length (Figure 5B). Individual bacteria in treated cultures were measured and compared with those from the non-irradiated sample (NI). The average size of at least 200 cells in each experiment was obtained, and it was observed increase in length with higher UV-C doses (Figure 5C). Statistically, these data sets display significant difference, with P_value_<0.001. To better compare the elongated population among the different treatment conditions, the bacterial length values were categorized. Length values corresponding to each of the NI culture quartiles were used to determine size categories. The fraction of persistent cells from the irradiated cultures that were included in the longest category changed from 25% in NI to 46% in the lower UV-C dose, and to 61% in the higher dose (Figure 5C). This is the first observation of filamentation in *L. interrogans* following the stress induced by DNA damage.

### Depletion of LexA repressors following UV-C irradiation

**Figure 6:**
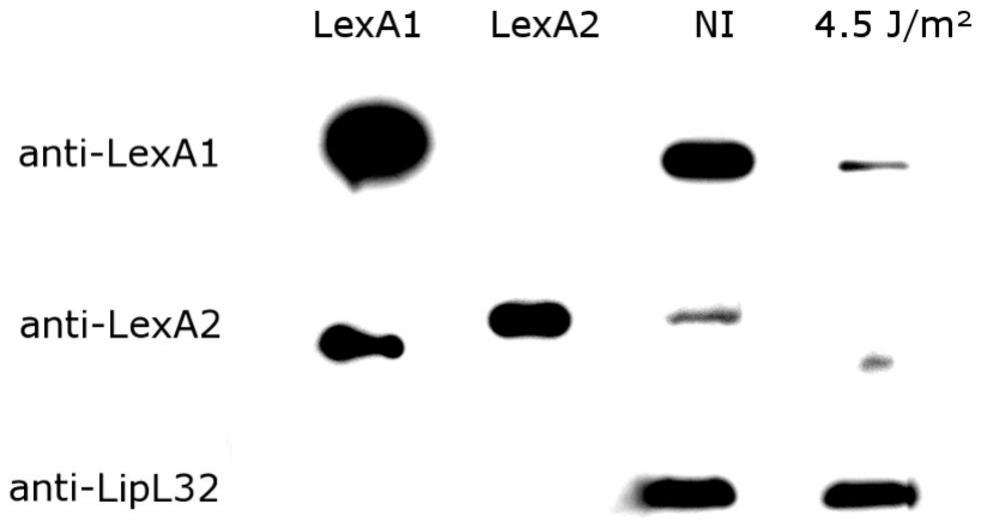
Presence of LexA1 and LexA2 repressors in *L. interrogans* extracts. Immunoblotting of total cell extract and purified LexA proteins was probed with antisera against LexA1 (1:5000 dilution), anti-LexA2 preincubated with 200 ng/µl LexA1 (1:1000 dilution) and anti-LipL32 (1:5000 dilution). The blots contained 20 ng of recombinant purified LexA1 and LexA2. In LexA1 and LipL32 blots, 40 µg of cell extracts from non-irradiated (NI) or irradiated (4.5 J.m^-2^) cultures were used, whereas for detection of LexA2, 150 µg.

Filamentation of bacteria suggests the leptospiral cell division is arrested after UV-C treatment. To assess whether lepstospires are eliciting the SOS response to deal with this stress, the presence of intact LexA repressors in NI and irradiated cells with 4.5 J.m^-2^ was evaluated (Figure 6). For this, we produced recombinant LexA1 and LexA2 (Figure S3), as well as polyclonal antisera against these proteins. Anti-LexA1 serum was LexA1-specific, whereas anti-LexA2 showed some cross-reaction with recombinant purified LexA1 (Figure S4). The cells were harvested 12h after irradiation, since the expected duplication time for *L. interrogans* is 8-12h [42]. Both proteins were detected by western blot in the non-irradiated culture, though LexA1 was barely detectable in the irradiated extract, while LexA2 was not (Figure 6). The lower band is presumably one of the LexA2 fragments resulting from its cleavage. As a control, LipL32, an immunodominant leptospiral lipoprotein [37], did not show changes after UV-C irradiation. These results suggest that both repressors are undergoing the self-cleavage responsible for SOS response induction.

### Some leptospiral genes potentially involved in DNA repair and lesion tolerance are UV-C inducible

**Figure 7:**
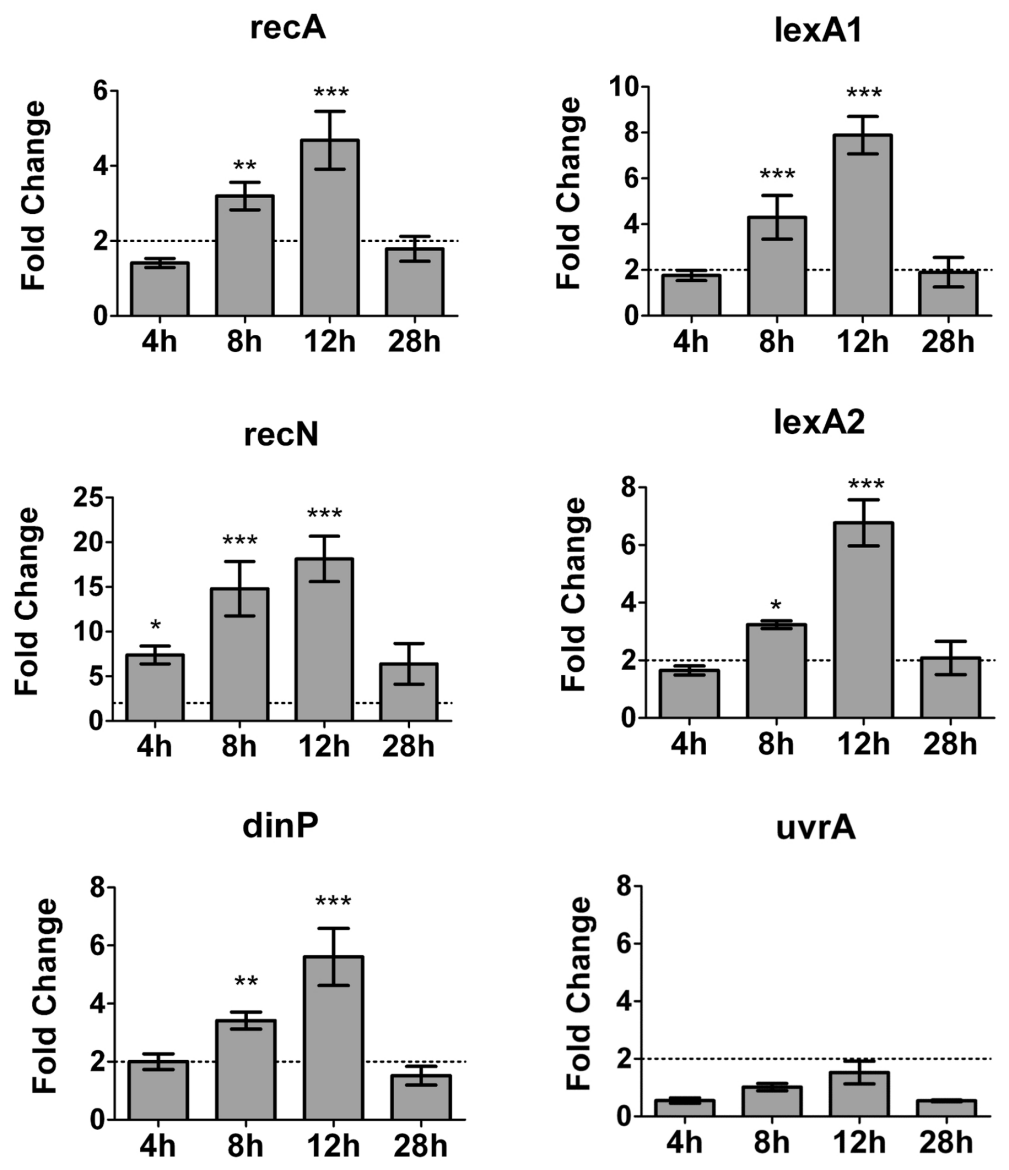
Expression kinetics of *L. interrogans* serovar Copenhageni genes in response to UV-C. The *Leptospira* culture was irradiated with 4.5 J.m^-2^, kept in the dark, and the RNA samples were obtained at the indicated time points. The fold change corresponds to gene expression in the irradiated sample *versus* the non-irradiated one at each time point, analyzed by qPCR. The 16S was used as normalizer. Error bars represent the standard deviation of the average of three independent experiments. Samples showing significant changes are indicated by *** (P value<0.001), ** (P value>0.01) or * (P value>0.05).

We searched for genes associated with LexA regulon and DNA damage stress induced by UV-C in *L. interrogans* serovar Copenhageni. The *recA* gene expression was examined in different times after UV-C exposure (Figure 7). The up-regulation of recA started 8h and reached its maximum 12h after UV-C exposure. The same assay was performed for *lexA1*, *lexA2* and other genes whose expression is generally UV-C regulated in other organisms (Table 1 and Figure 7): the recombination-involved gene *recN*, the DNA polymerase IV (*dinP/dinB*), responsible for translesion DNA synthesis and *uvrA*, the excinuclease subunit A from the nucleotide excision repair (NER). To avoid false positives, genes were considered differentially expressed only if their fold change following UV-C irradiation had strong statistic support. Except for the non-induced *uvrA*, all genes showed the same pattern of transcriptional regulation, with the difference that *recN* induction started sooner, after 4h. The similarity among the expression profiles is an indication of a co-regulation mechanism.

**Table 1:** Leptospiral genes tested for UV-C response.

ORF	Gene	Annotation	Putative SOS box	Fold Change
UV-C responsive*				
LIC11745	*recA*	Recombinase A	**TTTG**CTATA**CAAA**	6.28 ± 1.23
LIC11620	*recN*	DNA repair protein	**TTTG**TCTTC**CAAA**	19.85 ± 0.47
LIC12305	*lexA1*	LexA repressor	**TTT**ATTCTTA**AAA**	8.38 ± 1.78
LIC12304		hypothetical protein		9.34± 1.31
LIC12303		hypothetical protein		2.80± 0.26
LIC12654	*lexA2*	LexA repressor	**TTT**AAATGT**CAA**G	6.76 ± 1.96
LIC12653		hypothetical protein		6.81 ± 1.58
LIC12655		hypothetical protein		5.65 ± 1.64
LIC13052	*dinP*	DNA polymerase IV	**TT**C**G**AAATTG**AAA**	5.60 ± 2.20
Non UV-C responsive*				
LIC10265	*ISlin1*	Transposase	**TTTG**GCGAT**CAAA**	0.69 ± 0.04
LIC10344	—	Anti-sigma factor antagonist	**TTTG**ATTTC**CAAA**	0.52 ± 0.06
LIC10362	—	Hypothetical protein	**TTTG**CTCTC**CAAA**	0.58 ± 0.19
LIC10491	*acrB*	Acriflavin resistance	**TTTG**TGTTT**CAAA**	0.31 ± 0.10
LIC10647	—	SoxW family	**TTTG**AAGAT**CAAA**	0.80 ± 0.09
LIC10867	—	CopG/DNA-binding domain	**TTTG**CTTCG**CAAA**	0.46 ± 0.06
LIC10881	—	TonB dependent porin	**TTTG**AAAGT**CAAA**	0.73 ± 0.83
LIC11702	*dnaG*	DNA primase	**TTTG**TTGGA**CAAA**	0.58 ± 0.11
LIC11925	—	Terminase GpA (phage)	**TTTG**CGATT**CAAA**	0.83 ± 0.18
LIC12993	—	High affinity receptor for IgE Fc	**TTTG**TTTTT**CAAA**	0.52 ± 0.21
LIC13395	—	OsmC-like protein	**TTTG**GACAT**CAAA**	1.03 ± 0.41
LIC11717	*uvrA*	Excinuclease ABC subunit A	—	1.26 ± 0.72
LIC12941	*uvrB*	Excinuclease ABC subunit B	—	0.84 ± 0.48
LIC11756	*uvrC*	Excinuclease ABC subunit C	—	1.49 ± 0.64
LIC11624	*uvrD*	Excinuclease ABC subunit D	—	1.53 ± 0.19
LIC11148	*ruvA*	DNA helicase subunit A	—	1.45 ± 0.08
LIC12811	*ruvB*	DNA helicase subunit B	—	1.32 ± 0.76
LIC12885	*ruvC*	Endodeoxyribonuclease	—	1.92 ± 1.00
LIC12112	*ssb*	Single-stranded DNA binding protein	—	0.58 ± 0.15
LIC13064	*tag*	3-methyladenine DNA glycosylase I	—	0.96 ± 0.91
LIC12362	*alkA*	3-methyladenine DNA glycosylase	—	1.01 ± 0.66
LIC11759	*nth*	Endonuclease III	—	0.43 ± 0.21
LIC10016	*maf*	Cell division Inhibitor	—	1.15 ± 0.89
LIC10837	*sulA*	Cell division Inhibitor	—	0.71 ± 0.06
LIC12245	*hfq*	Host factor-1 (RNA degradation)	—	0.47 ± 0.17
LIC12017	*clpB*	ATP-dependent protease	—	0.34 ± 0.08
LIC10222	*dnaE*	DNA polymerase III subunit alpha	—	0.69 ± 0.13
LIC12109	*dnaB*	Replicative DNA helicase	—	0.61 ± 0.19
LIC11339	*phr*	Deoxyribodipyrimidine photolyase	—	0.72 ± 0.25

Fold change is relative to expression in the non-irradiated sample, and 16S gene was used as normalizer. The *recA* gene SOS box palindrome is indicated in bold letters.

* After one-way ANOVA and Dunnet post-test, genes with P_value_ < 0.001 were considered UV-C responsive.

In an attempt to identify other genes that could be required following UV-C induced stress, 21 genes involved in DNA repair and members of the SOS regulon in other organisms [43-45] had their expression levels measured. None of the new genes tested was induced by UV-C (Table 1). Surprisingly, the homologues of two cell division inhibitor proteins, *sulA* from *E. coli* [46] and *maf*, from *B. subtilis* [47] did not respond to UV-C. In addition, a bioinformatic search was performed for the described SOS box palindrome **TTTG**N _5_ **CAAA** from *L. interrogans* serovar Lai [25] in the upstream region of all annotated genes in the genome of *L. interrogans* serovar Copenhageni. Seventy genes were detected, including nine identical ISlin1 transposases (Table S3). As expected, the exact palindrome was found in *recA* promoter region, in addition to *recN*, and genes encoding some permeases, oxidoreductases, and others. From this set, 11 genes were tested by qPCR, but also failed to increase their expression after UV-C irradiation (Table 1).

### Analyses of *lexA1 and lexA2* vicinities reveal other UV-C inducible genes

**Figure 8:**
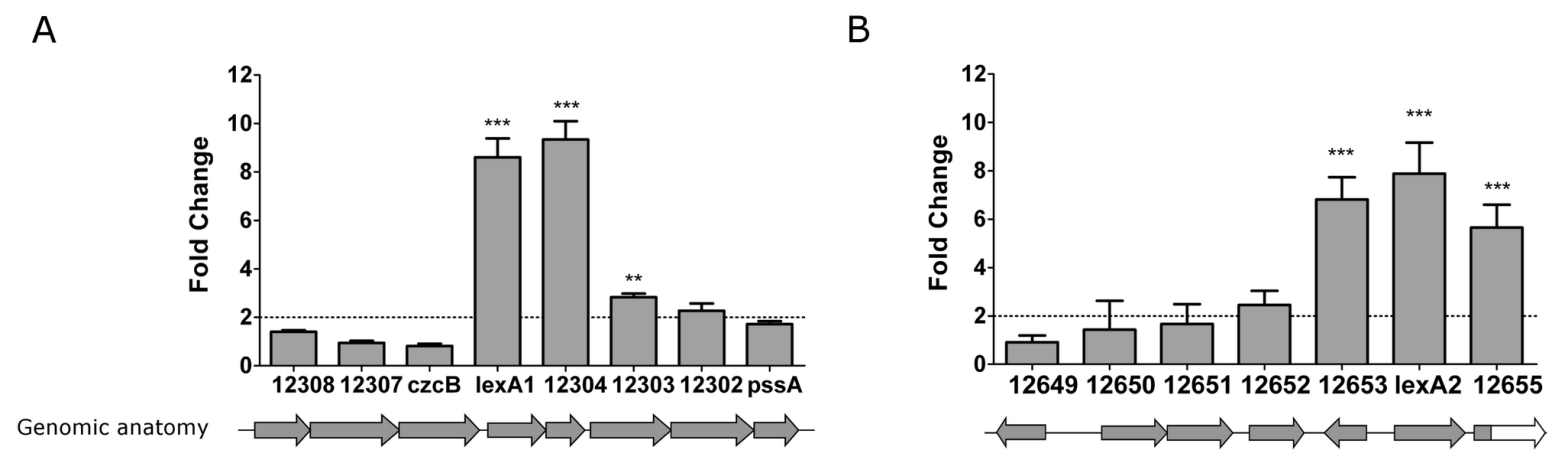
Expression of transcripts in (A) *lexA1* and (B) *lexA2* genomic regions post-treatment with UV-C. The graphic represents the fold change in gene expression in the irradiated sample *versus* the non-irradiated one 12h after irradiation with 4.5 J.m^-2^ and analyzed by qPCR. Genes showing significant changes are indicated by *** (P value<0.001) or ** (P value>0.01). Below, the genomic organization is indicated.

The gene expression pattern after UV-C exposure was also evaluated for the *lexA1* and *lexA2* genomic regions. Interestingly, the genes in *lexA1* operon exhibits distinct regulation pattern after UV-C treatment. While *lexA*1 and LIC12304 showed more than eight-fold increase in expression, LIC12303 barely reached the cutoff (Figure 8A). The other genes did not display significant expression change in irradiated sample relative to the non-irradiated one. Thus, this profile suggests the presence of an alternative promoter within the *czcB*-*lexA1* intergenic region, which may be bound by LexA1, in contrast with previous findings [25]. In the case of *lexA2*, we have already shown that the six genes in the vicinity of *lexA2* are probably expressed in three different transcripts (Figure 4). These transcriptional units became more evident during analysis of induction after UV-C irradiation by qPCR (Figure 8B). The LIC12649 and LIC12650-12652 transcripts did not show significant fold change in expression, whereas LIC12653 and *lexA2*-LIC12655 were both up regulated by more than six fold (Figure 8B). Since these genes are oriented in opposite directions, they probably share an SOS box.

### Correlation between UV-C-up-regulation and LexA1 binding

**Figure 9:**
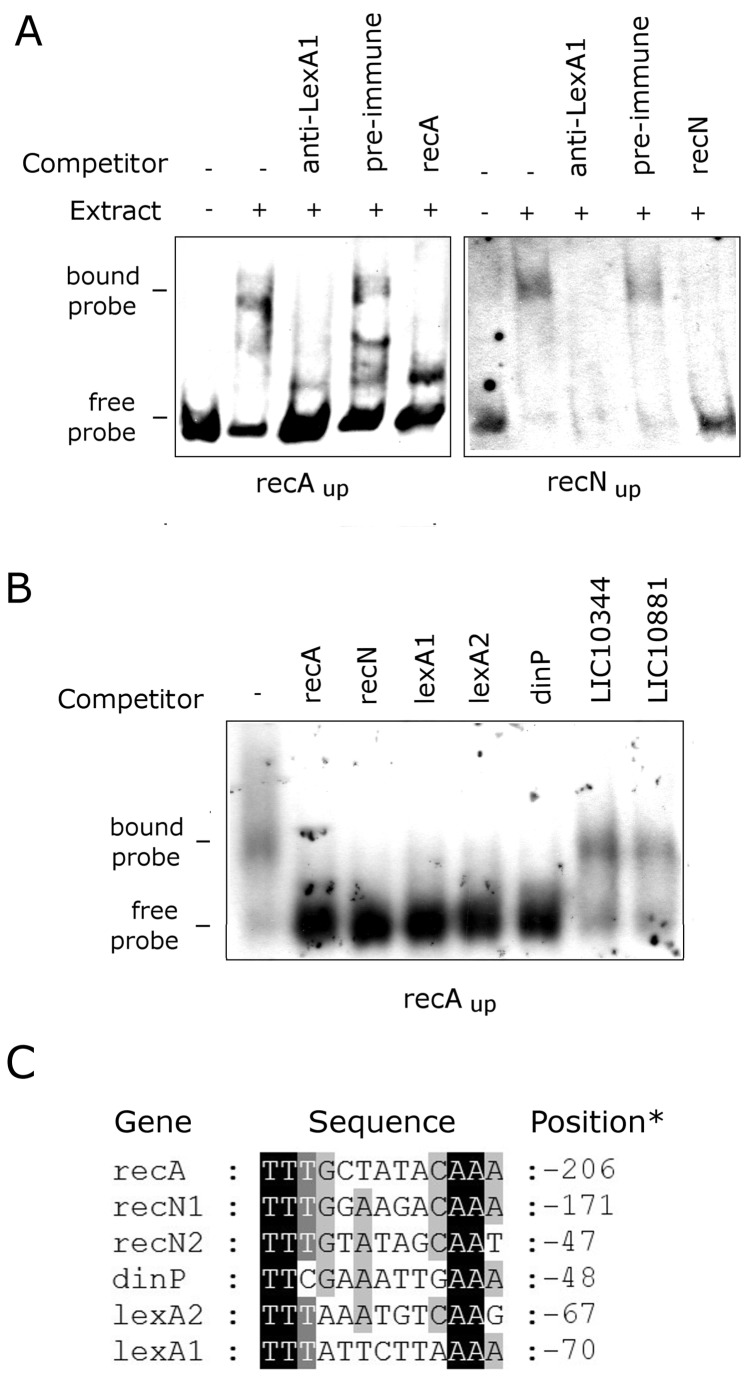
Analysis of LexA1 binding. (A) LexA1 binding assays (40 µg cell extract) with 1.55 fmol DIG-labeled probes corresponding to the upstream sequences of *recA* (recA_up_) and *recN* (recN_up_). Competitors were anti-LexA1 or pre-immune sera (1 µl each), as well as non-labeled probes in 200 fold excess to the labeled ones. Bound and free labeled probes are indicated. (B) LexA1 competition assays, done as in (A), but with 1.55 fmol [γ-^32^P] ATP-labeled recA_up_. After the binding reaction with protein extracts (40 µg), 200 fold excess of non-labeled fragments were added, corresponding to the upstream sequences of the indicated genes. (C) Alignment of the putative SOS box sequences from UV-C-induced promoter genes. * Distance of the central nucleotide in the palindrome to the initial codon.

We performed EMSA to test whether the recombinant LexA1 was able to bind to the UV-C up-regulated gene promoters, using the *recA* promoter as positive control (recAup). As purified LexA1 did not show DNA-binding activity (data not shown), we tested if total cell extract contains any factor with this activity (Figure 9A). The incubation of *recA* and *recN* promoters with leptospiral extract revealed the formation of protein/DNA complex, as mobility shifts were obtained (Figure 9A). The specificity of the protein/DNA complex was confirmed by competition with 200 fold excess of unlabeled DNA fragment identical to the probe binding assay, resulting in abolishment of the complex (Figure 9A). To ascertain if LexA1 was present in the protein/DNA complex, we incubated the extract with anti-LexA1 prior to the addition of the probe (Figure 9A). The formation of the complex was completely abolished, indicating the recognition of LexA1 by the antibody, whereas pre-immune serum was not able to inhibit binding. This result confirms the binding of LexA1 to two of the UV-C induced gene promoters. Our analysis proceeded with competition binding experiments to address if LexA1 also binds to *lexA1*, *dinP* and *lexA2* promoters, as they were UV-C induced (Figure 9B). To decrease background problems, [γ-^32^P] ATP-labeled *recA* promoter was used as probe. The LexA1/recAup complex was disrupted when unlabeled DNA fragments containing the promoters from *recA*, *recN*, *lexA1*, *lexA2* and *dinP* were added to the binding reaction in 200-fold excess. Promoters from all UV-C up-regulated genes were extremely effective in abolishing the protein/DNA complex in this concentration. On the other hand, sequences containing the previously predicted LexA1 box upstream of non UV-C-induced genes did not abolish the complex. It is important to emphasize that total cell extract contains many different DNA-binding proteins, and it is possible to have non-specific binding to the DNA probe. In these situations, the protein/DNA non-specific complexes migrate very slowly and diffusely, and are not abolished by the specific competitor [48]. Therefore, the complexes disrupted in our EMSAs are probably specific to LexA1 protein.

The exact SOS box described for serovar Lai [25] was found only in the promoter regions of *recA* and *recN*. However, our results point to LexA1 binding to its own promoter, as well as *lexA2* and *dinP* upstream sequences, suggesting that there is some flexibility of sequence for LexA1 binding. Alignment of these promoters revealed the presence of similar versions of the TTTGN _5_CAAA palindrome in all of the UV-C induced genes (Figure 9C). The *lexA1* gene has a modified SOS motif, TTTAttcttAAAA. This palindrome could be responsible for gene repression, even with two mismatches. The same could be true for *dinP*, which harbors one possible SOS motive containing two substitutions, TTCGaaattGAAA. For *recN*, beyond the palindrome TTTGgaagaCAAA identified by our bioinformatics searches, there is a modified palindromic motif located within the gene predicted promoter: TTTGtatagCAAT . The alignment also shows that from the eight positions of the putative SOS box, only four are conserved: two thymines in one half and two adenines in the other half of the palindrome (Figure 9C).

### The *lexA2* promoter region is bound by both LexA repressors

**Figure 10:**
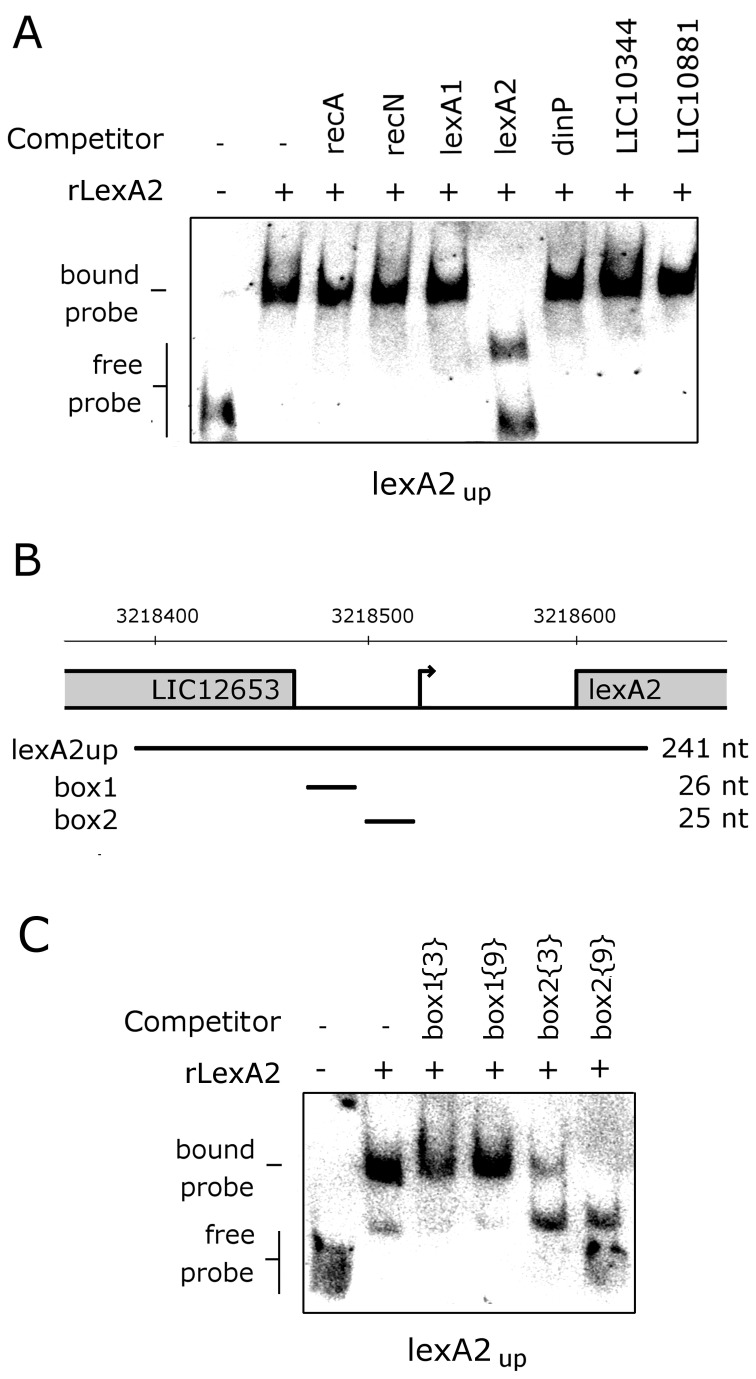
Analysis of LexA2 binding. (A) LexA2 binding assays were carried out with 80 ng of purified recombinant protein, using 1.55 fmol DIG-labeled lexA2_up_ as probe. For the competition assays, 200 fold excess of unlabeled probes was added to the binding reaction. (B) Scheme representing the fragments used for LexA2 binding experiments. Genomic coordinates and size of each fragment (in nucleotides) are indicated. Box 1 indicates the palindrome ATTCN _13_GAAT, and box 2, TTGTAN_10_TACAA. The putative *lexA2* promoter is indicated by an arrow. (C) Competition assays, where 200 fold excess of unlabeled probes was added to the binding reactions, corresponding to the two putative binding sites contained in lexA2up. The number of nucleotides flanking the palindromes are indicates in braces ({3} or{9}).

Gel mobility shift assays were done using purified recombinant LexA2 to test its ability to bind to promoter sequences of UV-C induced genes. The recombinant protein was incubated with labeled DNA segment of its own promoter (lexA2up), and the LexA2/DNA complex was retarded in the gel (Figure 10A). Formation of the upper complex was abolished by the presence of unlabeled upstream sequences from *lexA2*, but not from the other UV-C induced genes. Therefore, these results show the specific binding of LexA2 to its own promoter. The *lexA2* gene possesses a TTTAaatgtCAAG motif in its promoter region, possibly responsible for LexA1 binding. In addition, we identified two other palindromes, potential motifs for LexA2 binding: ATTCN _13_GAAT (box 1) and TTGTAN_10_TACAA (box 2) (Figure 10B). Formation of LexA2/lexA2up complex was not competed out by the ATTCN _13_GAAT motif. On the other hand, the fragment containing the TTGTAN_10_TACAA motif disrupted the complex formation (Figure 10C). A more efficient complex disruption was obtained with longer sequences flanking the palindrome. Accordingly, LexA2 probably binds to the motif **TTGTA**TGCAATGTCT**TACAA** , localized between -146 and -127 of *lexA2* coding sequence, adjacent to its putative promoter (-124 to -96) (Figure 10C).

## Discussion

The present work revealed that *L. interrogans* is capable of responding to DNA damage through a coordinated and reversible mechanism, the SOS system. In these situations, the triggered SOS response is not only involved in DNA repair, but also influences antimicrobial resistance spread, general stress response and induction of virulence factors in organisms as uropathogenic *E. coli*, *Salmonella enterica* and *Vibrio cholerae* [10,49,50]. These mechanisms probably also occur in *L. interrogans*, and the full characterization of the DNA damage response is the first step to identify them. The SOS induction in *L. interrogans* serovar Copenhageni after UV-C treatment was confirmed by LexA1 and LexA2 depletion 12h after UV-C exposure (Figure 6), probably a consequence of auto-proteolysis promoted by RecA. In addition, UV-C induced cell filamentation, as seen in several bacteria under stress [10]. This is the first documentation of *L. interrogans* filamentation induced by DNA damage. We could not determine the gene responsible for blocking the cell division, since the genes encoding both cell division protein orthologs present in *L. interrogans* serovar Copenhageni, *sulA* and *maf*, showed no increase in expression after UV-C exposure.

*L. interrogans* serovar Copenhageni has 10% survival after 18 J.m^-2^ (Figure 5A). For comparison, the serovar Pomona has this level of survival following exposure to approximately 7.5 J.m^-2^ [51], and the saprophytic *L. biflexa*, near 20 J.m^-2^ [52]. The obligatory pathogen *Borrelia burgdorferi* is even more sensitive and it displays 10% of survival with an UV-C dose of 8 J.m^-2^ [53]. According to these results, *L. interrogans* serovar Copenhageni has a relatively high resistance to UV-C, comparable to that showed by a free-living spirochete.

The *lexA2* gene is located in an ancient prophage-like region rich in genes encoding hypothetical proteins (Figure 4). Although some of these ORFs are also present in serovar Lai, *lexA2* seems to be exclusive to serovar Copenhageni (Figure S1 and Figure S2). Furthermore, our phylogenetic analysis points out to an event of horizontal gene transfer (Figure 2). Despite the differences in amino acid sequence, the predicted secondary structure of LexA2 shows the necessary features for its activity as a transcriptional repressor. The catalytic residues are correctly placed, as well as the scissile peptide bond, flanked by Cys and Gly (Figure 1). This is not the usual Ala-Gly scissile peptide bond, found in all characterized LexA proteins. However, this substitution still allowed the *E. coli* LexA repressor to undergo autolysis, probably due to similar sizes of side chains in alanine and cysteine [54].

The best way to investigate the role of LexA2 would be the characterization of *lexA1* and *lexA2* mutants. However, *L. interrogans* serovar Copenhageni remains one of the serovars most refractory to genetic transformation. Nevertheless, since only a minor proportion of the DNA transferred from an organism to another is likely to be established in the recipient genome [55], the maintenance of *lexA2* in *L. interrogans* serovar Copenhageni suggests it may have an important role in the bacterium.

The consequence of RecA-dependent proteolysis of LexA is the de-repression of SOS-regulated genes [11]. In this work we identified nine genes that were upregulated following UV-C treatment. They are divided in six transcriptional units (*lexA1-*LIC12304, LIC12303, LIC12653, *lexA2-*LIC12655, *recA, recN* and *dinP*) (Figure 8 and Table 1). All of them presented the same pattern of expression, and their mRNA levels reached a maximum 12h after irradiation. The timing agrees with the expected duplication time, since the attempt of DNA replication is necessary to activate RecA [56]. Twenty-eight hours after the induction the system returned to its normal configuration and no increase on transcripts levels was observed (Figure 7). This coincident pattern of expression corroborates the hypothesis of these genes sharing the same transcriptional control.

In our hands, purified LexA1 protein was unable to bind to promoter sequences of UV-C-induced genes. Thus, we used crude cell extracts in our EMSAs (Figure 9). This approach tries to overcome the problems of working with cell-free systems, where the choice of buffer, component concentrations and lack of additional proteins could limit the ability to detect potential LexA1 binding sites [57]. Our results show the formation of protein/DNA complex for *recA* and *recN* promoters (Figure 9A). An antibody blockage reaction confirmed that LexA1 was responsible for the observed shifts. Preincubation of the protein with a specific antibody favors the inhibition of DNA/protein complex formation, leading to the disappearance of the shifted band rather than supershifting it [58]. We used competition binding experiment to address if LexA1 also binds to the promoters of the remaining UV-C-induced genes (*lexA1*, *lexA2* and *dinP*). The disruption of LexA1/recAup complex confirmed the binding affinity of LexA1 to the corresponding upstream sequences (Figure 9B). Therefore, we concluded that LexA1 has binding affinity to the promoters of UV-C-induced genes. The *L. interrogans* serovar Copenhageni LexA1 regulon includes at least genes involved in DNA repair (*recA* and *recN*), DNA damage tolerance (*dinP*), both *lexA* orthologs and four genes encoding hypothetical proteins. The previous SOS motif identified for *Leptospira* LexA [25] is found upstream of *recA* and *recN* genes. However, degenerated sequences are found upstream of *lexA1*, *dinP* and *lexA2* genes (Figure 9C), suggesting that there is some flexibility of sequence for LexA1 binding.

The expression analysis after UV-C treatment in *lexA1* genomic region suggests the presence of an additional internal promoter, upstream of *LexA1*, since only *lexA1* and the downstream genes LIC12304 and LIC12303 were upregulated (Figure 8A). Therefore, this gene cluster may contain additional regulatory sequences, generating transcripts of various lengths. In addition, the hypothetical transcriptional regulator LIC12304 may control other genes, increasing the response complexity.

The *lexA2* gene was also induced by UV-C irradiation (Figure 7), in addition to LIC12655 and LIC12653 (Figure 8B). While LIC12655 encodes a putative M28 peptidase, LIC12653 encodes a hypothetical protein. LexA2 has the ability of binding to its own promoter region, recognizing a SOS box different than that recognized by LexA1, indicated by the EMSA experiments (Figure 10). This could be a result of the different amino acid composition in their respective DNA binding domains (Figure 1). Similarly to what was observed in this work, *Xanthomonas axonopodis* LexA1 binds to *lexA1* and *lexA2* promoters, but LexA2 binds only to its own promoter [59]. This divergences may be consequence of relaxed selection of the extra regulators, and it is probably associated with very small regulons in these cases [60].

Although *recN* was not initially described as a potential member of the leptospiral SOS regulon, it possibly harbors two SOS boxes: the same palindrome found at *recA* promoter, and another one containing a mismatch (Figure 9C). Therefore, this gene may be tightly regulated through multiple binding sites. Leptospiral *recN* expression is induced by UV-C before any other gene analyzed, and reaches almost 20 fold increase after 12h of UV-C treatment (Figure 7 and Table 1). In addition, RecN and RecA may have important roles in the maintenance of virulence, since in *L. interrogans* serovar Lai their expression is enriched in a virulent strain when compared to a virulence-attenuated one [61].

Surprisingly, various genes involved in DNA repair were not UV-C induced (Table 1). Among the *uvr* group (*uvrA, -B, -C, -D*), *uvrA* usually belongs to the SOS regulon, as well as *ruvAB* and *ssb*, although their repression by LexA is not unanimous through different taxa [8]. The genes encoding for proteins involved in Base Excision Repair (BER), responsible for removing small, non-helix-distorting base lesions from the genome, were not induced as well (Table 1). Possibly the basal level of expression of these genes is sufficient to maintain the genome integrity, which would explain the lack of UV-C induction. Taking into account the *L. interrogans* lifestyle, and the amount of environmental pressure this bacterium undergoes either inside or outside the host, a constitutive expression of Nucleotide Excision Repair (NER), a very flexible and versatile DNA repair pathway that removes helix distorting lesions from the genome, and BER genes makes sense [62]. In addition, the recombination repair (which includes RecA and RecN) may play a major role in DNA damage response in leptospires, dealing with the additional DNA lesions generated after UV-C treatment.

Trying to extend the known SOS regulon of *L. interrogans*, we performed a search for the TTTGN _5_CAAA palindrome [25] in the upstream regions of the entire *L. interrogans* serovar Copenhageni genome (Table S3). Some of the resulting genes had their expression levels tested after UV-C irradiation, including one of the nine identical ISlin1 transposases. The SOS response is known to regulate several transposable elements, such as integrons and transposons [63,64]. Interestingly, none of the tested genes exhibited increase in their expression after UV-C irradiation (Table 1). One explanation for this difference is that sequences outside the operator region may affect the interaction of LexA with operator bases *in vivo*. A similar situation was observed for LexA repressor from *B. subtillis* [65]. Also, we cannot exclude the possibility of an accessory protein playing a role in the LexA1/promoter complex formation.

In *M. tuberculosis*, it was found that LexA was capable of binding to sites with up to three mismatches to the original SOS box [66]. *Leptospira* LexA1 may be capable of binding to the degenerated sequences through the interaction with other proteins present in the extract. The sequence originally considered here is the one present specifically at the *recA* operator [25], though the EMSA results (Figure 9A) show that LexA1 repressor is also capable of binding to somewhat different palindromes. SOS boxes from some organisms are rather variable, as for *Petrotoga miotherma*, in which the consensus GANTN _6_ GANNAC permits a variety of binding sequences [26]. This may be the case of *L. interrogans* serovar Copenhageni. We have few genes to evaluate a new SOS box, but it is possible that nucleotides positioned adjacent to the palindrome, or inside the spacer, play an important role in LexA1 binding.

In this study, we expanded the knowledge on the DNA damage response of *L. interrogans* serovar Copenhageni and on the SOS regulon. UV-C exposure caused the up-regulation of at least nine genes, including *lexA1* and *lexA2*, a second lexA repressor also involved in the SOS response. We were able to show a correlation between the UV-C-dependent increase in the expression of these genes and LexA1 binding to their upstream sequences by EMSA. The depletion of LexA1, as a consequence of self-cleavage triggered by DNA damage, would release it from the promoters and allow the accessibility of RNA polymerase apparatus. Moreover, *lexA2* promoter is also bound by LexA2. In this regard, we were able to characterize the motif (TTGTAN_10_TACAA) responsible for LexA2 binding at the *lexA2* promoter. The existence of two functional LexA repressors indicates a more complex DNA damage response in leptospires than previously imagined. Still, a more specific approach is needed to identify the definitive leptospiral SOS box, and other LexA1 or LexA2 regulated genes.

## Supporting Information

Figure S1 **Comparison of *lexA2* region between sequenced Leptospiras.** Scheme representing MEGABLAST searches against Leptospiras genome sequence projects used the whole genome shotguns contigs database (wgs) at GenBank. The regions shown (red strands) are those with alignment score greater than 200, relative to the region in *L. interrogans* serovar Copenhageni.
(PDF)

Figure S2 **Presence of *lexA1* and *lexA2* in the genome of different leptospires detected by PCR.** The reactions used 20ng of genomic DNA, and the primers were designed for serovar Copenhageni, according to Table 1. The negative reaction was carried without template DNA. The upper panel corresponds to *lexA1*, while lower panel, to *lexA2*. All amplicons had the expected molecular size corresponding to 621 bp for *lexA1* and 630 bp for *lexA2*.
(PDF)

Figure S3 **Expression and purification of 6xHis tagged recombinant LexA1 and LexA2.** Both proteins were expressed in soluble form in *E. coli* BL21(DE3) Star pLysS after IPTG-induction. The soluble fractions of the extracts were clarified by filtration and used as column input. After washes, the proteins were eluted by 400 mM imidazole.
(PDF)

Figure S4 **Titration and specificity of anti-LexA1 and -LexA2 sera.** Anti-sera were generated by intraperitoneal immunization of five BALB/c mice with 10 µg of purified protein in Al(OH)_3_. The immunizations were performed weekly in four doses and mice were bled by the retrorbital plexus one week after the last dose. (A) Sera titration following the protocol by Hauk et al. (2005), comparing pre-immune and immune sera. Continuous line with squares corresponds to anti-LexA1, while discontinuous line with circles corresponds to anti-LexA2; triangles mark the pre-immune serum. (B) Cross-reaction analyses. The continuous line represents anti-LexA1, and the discontinuous one represents anti-LexA2. Squares stand for coating with purified LexA1, and circles, LexA2.
(PDF)

Table S1 **LexA amino acid sequences used for phylogenetic analysis.** It is shown the complete species name for each code used in Figure 2, with phylum classification, in addition to the GenBank accession number of the correspondent protein sequences.
(PDF)

Table S2 **Oligonucleotides used in this study.**
(PDF)

Table S3 **Presence of the TTTGN _5_CAAA palindrome in upstream sequences of *L. interrogans* serovar Copenhageni genes.**
(PDF)
