# Supplementary material for: Leptospira interrogans serovar Copenhageni Harbors Two lexA Genes Involved in SOS Response
Source: PLoS One. 2013 Oct 3;8(10):e76419. doi: 10.1371/journal.pone.0076419 (PMC3789691; doi:10.1371/journal.pone.0076419)
Supplement: Figure S1 — Comparison of lexA2 region between sequenced Leptospiras. Scheme representing MEGABLAST searches against Leptospiras genome sequence projects used the whole genome shotguns contigs database (wgs) at GenBank. The regions shown (red strands) are those with alignment score greater than 200, relative to the region in L. interrogans serovar Copenhageni. (PDF) [file pone.0076419.s001.pdf]

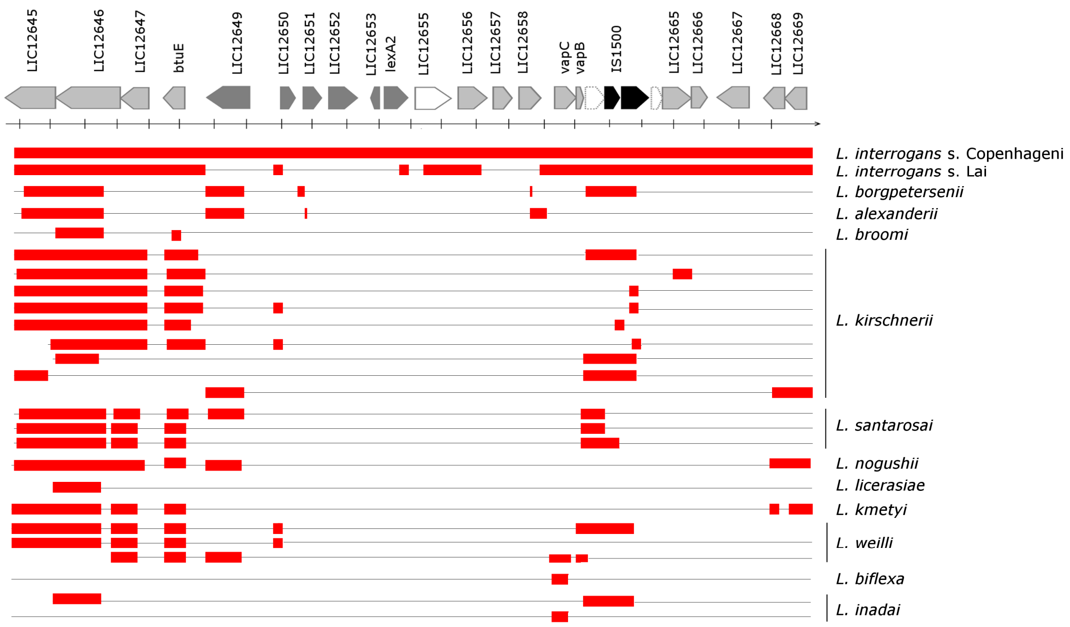


**Figure S1. Comparison of *lexA2* region between sequenced Leptospiras.** Scheme representing MEGABLAST searches against Leptospiras genome sequence projects used the whole genome shotguns contigs database (wgs) at GeneBank. The regions shown (red strands) are those with alignment score greater than 200, relative to the region in *L. interrogans* serovar Copenhageni.
