# Supplementary material for: Leptospira interrogans serovar Copenhageni Harbors Two lexA Genes Involved in SOS Response
Source: PLoS One. 2013 Oct 3;8(10):e76419. doi: 10.1371/journal.pone.0076419 (PMC3789691; doi:10.1371/journal.pone.0076419)
Supplement: Figure S2 — Presence of lexA1 and lexA2 in the genome of different leptospires detected by PCR. The reactions used 20ng of genomic DNA, and the primers were designed for serovar Copenhageni, according to Table 1. The negative reaction was carried without template DNA. The upper panel corresponds to lexA1, while lower panel, to lexA2. All amplicons had the expected molecular size corresponding to 621 bp for lexA1 and 630 bp for lexA2. (PDF) [file pone.0076419.s002.pdf]

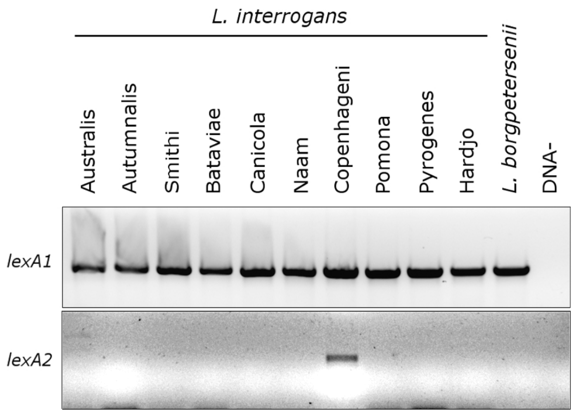

Figure S2. Presence of *lexA1* and *lexA2* in the genome of different leptospires detected by PCR. The reactions used 20ng of genomic DNA, and the primers were designed for serovar Copenhageni, according to Table 1. The negative reaction was carried without template DNA. The upper panel corresponds to *lexA1*, while lower panel, to *lexA2*. All amplicons had the expected molecular size corresponding to 621 bp for *lexA1* and 630 bp for *lexA2*.
