# Supplementary material for: Leptospira interrogans serovar Copenhageni Harbors Two lexA Genes Involved in SOS Response
Source: PLoS One. 2013 Oct 3;8(10):e76419. doi: 10.1371/journal.pone.0076419 (PMC3789691; doi:10.1371/journal.pone.0076419)
Supplement: Figure S3 — Expression and purification of 6xHis tagged recombinant LexA1 and LexA2. Both proteins were expressed in soluble form in E. coli BL21(DE3) Star pLysS after IPTG-induction. The soluble fractions of the extracts were clarified by filtration and used as column input. After washes, the proteins were eluted by 400 mM imidazole. (PDF) [file pone.0076419.s003.pdf]

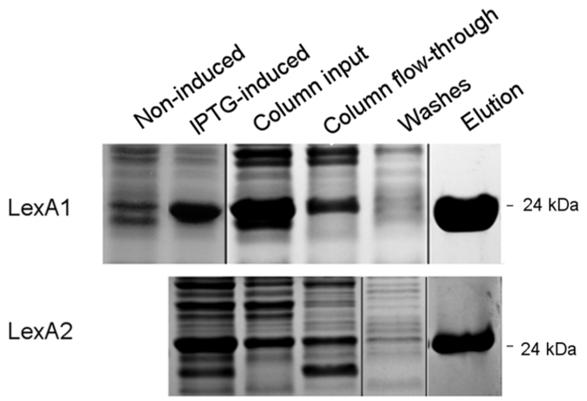

FigureS3. Expression and purification of 6xHis tagged recombinant LexA1 and LexA2. Both proteins were expressed in soluble form in *E. coli* BL21(DE3) Star pLysS after IPTG-induction. The soluble fractions of the extracts were clarified by filtration and used as column input. After washes, the proteins were eluted by 400 mM imidazole.
