# Supplementary material for: Leptospira interrogans serovar Copenhageni Harbors Two lexA Genes Involved in SOS Response
Source: PLoS One. 2013 Oct 3;8(10):e76419. doi: 10.1371/journal.pone.0076419 (PMC3789691; doi:10.1371/journal.pone.0076419)
Supplement: Figure S4 — Titration and specificity of anti-LexA1 and -LexA2 sera. Anti-sera were generated by intraperitoneal immunization of five BALB/c mice with 10 µg of purified protein in Al(OH)3. The immunizations were performed weekly in four doses and mice were bled by the retrorbital plexus one week after the last dose. (A) Sera titration following the protocol by Hauk et al. (2005), comparing pre-immune and immune sera. Continuous line with squares corresponds to anti-LexA1, while discontinuous line with circles corresponds to anti-LexA2; triangles mark the pre-immune serum. (B) Cross-reaction analyses. The continuous line represents anti-LexA1, and the discontinuous one represents anti-LexA2. Squares stand for coating with purified LexA1, and circles, LexA2. (PDF) [file pone.0076419.s004.pdf]

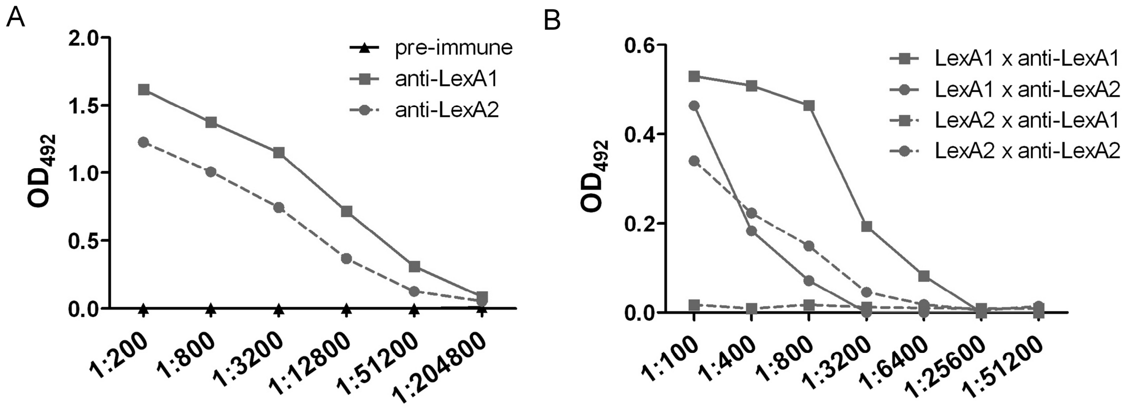

Figure S4. Titration and specificity of anti-LexA1 and -LexA2 sera. Anti-sera were generated by intraperitoneal immunization of five BALB/c mice with 10 µg of purified protein in $Al(OH)_3$. The immunizations were performed weekly in four doses and mice were bled by the retrorbital plexus one week after the last dose. (A) Sera titration following the protocol by Hauk *et al.* (2005), comparing pre-immune and immune sera. Continuous line with squares corresponds to anti-LexA1, while discontinuous line with circles corresponds to anti-LexA2; triangles mark the pre-immune serum. (B) Cross-reaction analyses. The continuous line represents anti-LexA1, and the discontinuous one represents anti-LexA2. Squares stand for coating with purified LexA1, and circles, LexA2.
