# Supplementary material for: Leptospira interrogans serovar Copenhageni Harbors Two lexA Genes Involved in SOS Response
Source: PLoS One. 2013 Oct 3;8(10):e76419. doi: 10.1371/journal.pone.0076419 (PMC3789691; doi:10.1371/journal.pone.0076419)
Supplement: Table S1 — LexA amino acid sequences used for phylogenetic analysis. It is shown the complete species name for each code used in Figure 2, with phylum classification, in addition to the GenBank accession number of the correspondent protein sequences. (PDF) [file pone.0076419.s005.pdf]

**Table S1. LexA amino acid sequences used for phylogenetic analysis.** It is shown the complete species name for each code used in Fig. 2, with phylum classification, in addition to the GenBank accession number of the correspondent protein sequences.

| Code | Species | Phylum | GenBank |
|---|---|---|---|
| E.coli | *Escherichia coli* K12 | Proteobacteria | NP_418467 |
| L.biflexa | *Leptospira biflexa* serovar Patoc strain 'Patoc 1 (Paris) | Spirochaetes | YP_001838702 |
| L.intCop-LexA1 | *Leptospira interrogans* serovar Copenhageni str. Fiocruz L1-130 | Spirochaetes | YP_002239 |
| B.subtilis | *Bacillus subtilis* subsp. subtilis str. 168 | Firmicutes | NP_389668 |
| C.crescentus | *Caulobacter crescentus* CB15 | Proteobacteria | NP_420709 |
| L.intLai | *Leptospira interrogans* serovar Lai str. 56601 | Spirochaetes | NP_711628 |
| L.intHBL550 | *Leptospira borgpetersenii* serovar Hardjo-bovis L550 | Spirochaetes | YP_797527 |
| L.intHBJB6 | *Leptospira borgpetersenii* serovar Hardjo-bovis JB197 | Spirochaetes | YP_801257 |
| R.bacterium | *Alph proteobacterium* HTCC2255 | Proteobacteria | ZP_01450304 |
| A.pasteurianus | *Acetobacter pasteurianus* IFO 3283-01 | Proteobacteria | YP_003187623 |
| P.carbinolicus | *Pelobacter carbinolicus* DSM 2380 | Proteobacteria | YP_357152 |
| C.curtum | Cryptobacterium curtum DSM 15641 | Actinobacteria | YP_003151129 |
| T.neapolitana | *Thermotoga neapolitana* DSM 4359 | Thermotogae | YP_002535029 |
| C.novyi | *Clostridium novyi* NT | Firmicutes | YP_878184 |
| L.araneosa | *Lentisphaera araneosa* HTCC2155 | Lentisphaerae | ZP_01873579 |
| O.terrae | *Opitutus terrae* PB90-1 | Verrucomicrobia | YP_001820930 |
| P.acanthamoebae | *Parachlamydia acanthamoebae* str. Hall's coccus | Chlamydiae | ZP_06299261 |
| D.radiodurans | *Deinococcus radiodurans* R1 | Deinococcus-Thermus | NP_285667 |
| S.dysenteriae | *Shigella dysenteriae* 1012 | Proteobacteria | ZP_03064622 |
| E.fergusonii | *Escherichia fergusonii* ATCC 35469 | Proteobacteria | YP_002385147 |
| Meta6609893 | marine metagenome GOS_6609893 | -- | EBX29792 |
| Meta2899910 | marine metagenome GOS_2899910 | -- | ECV43343 |
| Meta2457973 | marine metagenome GOS_2457973 | -- | ECX88648 |
| S.helenicus | *Staphylothermus hellenicus* DSM 12710 | Crenarchaeota (Archaea) | YP_003669041 |
| L.weilii | *Leptospira weilii* str. 2006001855 | Spirochaetes | ZP_09264799 |
| Hyphomycrobium | *Hyphomicrobium* sp. MC1 | Proteobacteria | YP_004676088 |
| L.licerasiae | *Leptospira licerasiae* serovar Varillal str. MMD0835 | Spirochaetes | ZP_09260259 |
| L.noguchii | *Leptospira noguchii* str. 2006001870 | Spirochaetes | ZP_09261139 |
| L.santarosai | *Leptospira santarosai* str. 2000030832 | Spirochaetes | ZP_09254025 |
| P.entomophila | *Pseudomonas entomophila* L48 | Proteobacteria | YP_608022 |
| O.formigenes | *Oxalobacter formigenes* HOxBLS | Proteobacteria | ZP_04576339 |
| P.zucineum | *Phenylobacterium zucineum* HLK1 | Proteobacteria | YP_002130602 |
| G.sulfurreducens | *Geobacter sulfurreducens* PCA | Proteobacteria | NP_951103 |
| S.arenicola | *Salinispora arenicola* CNS-205 | Actinobacteria | YP_001536300 |
| P.mobilis | *Petrotoga mobilis* SJ95 | Thermotogae | YP_001568276 |
| C.hydrogenof | *Carboxydothermus hydrogenoformans* Z-2901 | Firmicutes | YP_360210 |
| L.araneosa | *Lentisphaera araneosa* HTCC2155 | Lentisphaerae | ZP_01873579 |
| C.akajimensis | *Coraliomargarita akajimensis* DSM 45221 | Verrucomicrobia | YP_003548426 |
| A.ferrooxidans | *Acidithiobacillus ferrooxidans* ATCC 53993 | Proteobacteria | YP_002219972 |
| S.caldaria | *Spirochaeta caldaria* DSM 7334 | Spirochaetes | YP_004699190 |
