# Supplementary material for: Leptospira interrogans serovar Copenhageni Harbors Two lexA Genes Involved in SOS Response
Source: PLoS One. 2013 Oct 3;8(10):e76419. doi: 10.1371/journal.pone.0076419 (PMC3789691; doi:10.1371/journal.pone.0076419)
Supplement: Table S2 — Oligonucleotides used in this study. (PDF) [file pone.0076419.s006.pdf]

Table S2. Oligonucleotides used in this study.

Correspondent Gene	Sequence Oligonucleotide Forward	Sequence Oligonucleotide Reverse	Amplicon (bp)
	For gene amplification (5' → 3')		
lexA1	TTGTTTAGTATGAAAGACCTG	GTAAATTCGATATAAACCG	618
lexA2	ATGAATAGGCAACGATCC	AGTATAAGACAATATTAACTTTAC	630
	For use in qPCR (5' → 3')		
16s	CATGCCGCGGTGAATACGTT	CGACTTCACCCCCTTCACGA	137
recA	TTGATTCTGTGGCGGCTTTGG	TCTAAGCGCCTGGGACATGAG	101
recN	GAGAATGTGGGCGGGATGGA	GTTGTGAACTTCGGCGAGCA	104
LIC12308	TTCTATCCCTCCCGAAAGT	TGGACCCGAGAAGAAATCA	150
LIC12307	ACCTTTGATTCTCCATTCTCCTCCTACC	GTAAACCGTCCTGACTTAGCTGCAC	123
czcB	CCCAAGCATTACCGAAGAAAG	CGACCCAGAAAGTATGGCTA	127
lexA1	TTGTGGCCTGAACGGGAAGT	GGCAAAGGGTGCCTACGATC	129
LIC12304	CGTTGATTCTTGCAATGCTTAC	AACTGAGACTTCGGACAGAG	149
LIC12303	GAAACCTCCATTCCCAATCCGC	CTCTATCAAGGAGCCATTCGAGGA	134
LIC12302	AACCAGGAGAAGAAGTCACAGCG	AGGGATTGTTCCAGAAATCGCCT	131
pssA	CATTCCATCTAATCCGTCGC	CTTACGCTCGGAAATCTCAC	147
LIC12649	CCAGGTGAACCGGATTCTACG	CGTATTCTGGAGCGGCACTT	154
LIC12650	CAAAGGTTATTGGTGCATCGTTCA	CACGCCGGTCAATAATCGGG	150
LIC12651	CGAAATCGAAGCAGTAATGAGCA	CCATTGTCGGTTTTGGCGTAGT	147
LIC12652	TTATTGCTCGCAGCGGCATG	TCCGATAACTCGGCTCCTTTCA	128
LIC12653	TCCGGATTCGATCTCGGGA	GTCAATGACTCGAGGTGAGTTTGT	105
lexA2	GGCGACCCGGTAGAACGAAT	TGCAATCACCAAGTCCCCATCT	131
LIC12655	ACTGCGGCGATTTTCGTAATTTTT	GGCACCTTGCTGTTTGCCAG	121
ruvA	CGGCGGAAGATTTGTATCGGA	AGCCGGACAAGAATAATTCCAGT	144
ruvB	GGGCAAGATCCGCTCCTTTG	CGCAAACGTGCACTGGATCA	145
ruvC	TGCGTCCCAAGAATCGTCGT	AAGGAACCACTGGAAGCGGA	107
uvrA	GTTTCACAACGTCCACCGCT	ACGCCGATCCGAGAGATGTT	101
uvrB	CCGTCGGTATGATACGCAGGA	ACGATCGAAAGGGACACGGT	137
uvrC	TCCAGGATCGTTGATCCCTTCG	AACCGGTTGCAAGTGGTGTG	101
uvrD	CCGTCATAAGTGGGAATACGTG	CATCGTCTCCAACCACACATAG	119
dinP	GAAACGCGCAAAATCATTCATGT	CAGCCGAAACAACCGATCTACTATT	133
sulA	TTATACAAGCAGCTTCTTTAGCCA	TCACTTTGCGAGAGTCAGGAAT	150
ssb	GGTATGTCGTCGTCACCACC	CTTCTGTTTCCTCTTCTCCAGCA	100
tag	GTAATCCAAGAAAGCCCAGCCT	AAACGTTGTGCTTGGGTTACCG	140
alkA	CCAGTTATGTTCGAGACCTTGC	CTACGGTCCAATTTCCAATCCC	132
nth	CTCGTTCATCCGTACATTGAGC	GCCTGATCCTGCGTTTCTAA	149
hfq	TCTTGGCGGGAATGATCGTAGAAATAGC	CTTGAAAGGCAAAGTGGTGAGCTTCG	110
maf	GGACCACAATCGTATCACAAGA	CGAATCAGAAGATATAGACGAAAGTTCT	134
dnaE	GGGATGTTGGACGAATACTTGG	CACGGGAAATACTCATCACCTG	136
dna	GTGGTTGAGTAACTACCGTGTC	CGGAGATCTTGTAGACAAACGG	143
phr	GCATACGATGGAAGAGACCCAA	GAATCGGAGGACATAGTTCGGA	136
clpB	ACGTAGTTCTCCTCTAGCAAGC	CGAGGTCAAATCTTCAGACGGA	122
LIC10254	ACTACGCTGGTTTGGTTCCG	CGCTTGAACAATCACCCTTCG	107
LIC13395	ACGATTCCTTTGATAGAACACATTCA	TGACAACTACATACAGAAGAAGCTAA	139
LIC10344	ATCCGGGCAGGGTGATATTT	ACAGGCGGAGTGATACAACA	142
dnaG	CTTTGCACCAGTTCCACCTT	GCCGGGAAACGGATAAACAA	104
LIC10362	TTGAAAGGGAGAATGAAGTGC	CAAAGACTCAAGAAACCGAAACA	134

acrB	GTGCCGGAGTTTCCATTTACCT	ACCGGAATTGCAGAAGTGATCG	122
LIC10647	CGTAAGAACGAATGGAGGTACTAAA	CGGGTAATCTTCGAGGTATGGA	116
LIC10867	CTCTAGTAAACTTGCGACCAACT	CTACGTGCGGCCAGTGAA	147
LIC10881	AACCAACGGGATTAACGTCG	TGGATTGAACCGCCTTGAGT	122
LIC11925	CCATCCATTTGTATCCGCAATTTCC	TAGTTTGTTTGGCGTGTGGC	111
LIC12993	CGGTTAAATCTCCGTAGTGTGC	CTCTCGTCACAAACCAATCTTCTG	135
LIC10107	TAAATGCGCCCAACGTGCTA	GTAGCCGCACTACTTTCTCCTT	144
LIC10382	ACTTCCTTCGCCTGCAACAA	TGCTCTTGCTTATCTCGCCA	148
pldB	CTGTGTTCTCCGATTCCGTGTT	AAGACGATACCTTTGCGGGA	118
LIC12708	TAACGGCTCTTAGTTGCACTCG	TGGAAGATTGGTTGGAACACCG	131
tas	GCACATAACTAGCATCAGGTGG	GTATGGGAACGATGACCTTTGG	138
LIC10767	GTTGCAGTATTTGTAGCCGTGGTGGT	GCCGCTTCTTCTGCCGTTCC	116
For use in RT-PCR (5' → 3')			
12309-12308	TCAAAGAAACGATTCGGTCTCTGGA	ACGGAAGGGGCTAAACGGTT	1302
12308-czcB	TCCCGTACTTAAAACCGCGAGA	ACGGAATGGGAGAAAGAACTTATCA	1594
czcB-lexA1	AGCCTGTTGTTTGTCTGTCAGG	AGCCTGTTGTTTGTCTGTCAGG	240
lexA1-12304	ACACTCTGTCCGAAGTCTCAGT	GGCAAAGGGTGCCTACGATC	541
12304-12303	TAAAACGAGAATGTGGATCTCCCAA	TCCGAAAAATCTCCGTCTGGAG	374
12303-12302	CCCTCCGTAGGGTTTATGTAAATCG	TTCAACCGGATGTAATTGTAAAGCC	444
12302-pssA	CCTGCTTCCGATGCTACGAG	GGAGGGGTGCTTGCAAACTT	298
pssA-12300	GGAGGGGTGCTTGCAAACTT	GCCTTACATCGCTTTTACGGTCA	1222
12650-12651	LIC12650 Forward (qPCR)	LIC12651 Reverse (qPCR)	1367
12651-12652	LIC12651 Forward (qPCR)	LIC12652 Reverse (qPCR)	703
12652-12653	LIC12652 Forward (qPCR)	LIC12653 Reverse (qPCR)	1351
12653-lexA2	LIC12653 Forward (qPCR)	lexA2 Reverse (qPCR)	742
lexA2-12655	lexA2 Forward (qPCR)	LIC12655 Reverse (qPCR)	866
For use in EMSA (5' → 3')			
recAup	GATTCTTTCTCAATAATACTCAAA	TGCTTTCTTCATGATACTTTCTCC	264
recNup	TCTAGAGGTTAAAGATATAGTAAG	TTCAGGGTCTTCAACATAC	267
lexA1up	CGCGCTCTTTGATGATTGCTGTGA	AGGAGATACCGTGGGAGATGAAGT	228
lexA2up	GACTTTCCTCTGGAAGGATCGTAG	GTTCATTTCTTCTGGATCGTTGCCT	242
dinPup	TACTACAGGCGCTTCTGCAAACGA	TCTCTTTGTTCTACGGAGGCA	225
10344up	CAGTTCGATTGAGTTCTCGAGGGT	GAGTGCTACCAGAGTATGTTTACCG	270
12993up	TCAATTTATTGACCTCGCATCT	ACTTGAATTTCTTTCTACGTTTTCG	254
