# Supplementary material for: Leptospira interrogans serovar Copenhageni Harbors Two lexA Genes Involved in SOS Response
Source: PLoS One. 2013 Oct 3;8(10):e76419. doi: 10.1371/journal.pone.0076419 (PMC3789691; doi:10.1371/journal.pone.0076419)
Supplement: Table S3 — Presence of the TTTGN 5CAAA palindrome in upstream sequences of L. interrogans serovar Copenhageni genes. (PDF) [file pone.0076419.s007.pdf]

Table S3. Presence of the TTTGN$_5$CAAA palindrome in upstream sequences of *L. interrogans* serovar Copenhageni genes.

	SOS box			
Gene	Start	End	Sequence	Annotation
LIC10107	-127	-115	tggaTTTGGGAACCAAAggga	peptidase s24, rhomboid family
LIC10265	-78	-66	cttcTTTGGCGATCAAAgcaa	transposase, ISlin1
LIC10344	-22	-10	catcTTTGGAAATCAAAacga	anti-sigma factor antagonist
LIC10362	-22	-10	aatcTTTGCTCTCCAAAaact	hypothetical protein
ivd	-90	-78	agttTTTGGAGAGCAAAgatt	isovaleryl-CoA dehydrogenase
LIC10382	-177	-165	ttacTTTGTTTTTCAAAtaaa	acyl-CoA dehydrogenase
LIC10450	-102	-90	ccatTTTGTTCGACAAAcacg	MerR family
acrB	-182	-170	cgccTTTGAGTTTCAAAaagg	acriflavine resistance
LIC10508	-238	-226	ttgaTTTGCCAAACAAAaaaa	M18 family
pldB	-224	-212	actcTTTGTTTTTCAAAatac	lysophospholipase
LIC10647	-204	-192	tgttTTTGATCTTCAAAaagg	SoxW family
LIC10695	-120	-108	gatgTTTGTTCCACAAAacat	hypothetical protein
LIC10712	-148	-136	ccttTTTGACGTGCAAAagtt	cytochrome C
LIC10742	-78	-66	cttcTTTGGCGATCAAAgcaa	transposase, ISlin1
LIC10767	-168	-156	aacaTTTGAAATACAAAggta	putative PTS permease protein
flaA-1	-227	-215	tggtTTTGTGCCTCAAAgtac	flagellar filament sheath protein
LIC10790	-144	-132	tgacTTTGAAAGGCAAAatta	hypothetical protein
rfe	-220	-208	cggaTTTGGCAATCAAAaacg	undecaprenyl-phosphate alpha-N-acetylglucosaminyltransferase
dapA	-22	-10	cgttTTTGATTGCCAAAtccg	dihydrodipicolinate synthase
LIC10867	-134	-122	gagtTTTGCTTCGCAAActtt	copG
LIC10881	-83	-71	gtaaTTTGACTTTCAAAaacg	TonB-dependent porin
LIC10886	-57	-45	cttcTTTGGCAATCAAAgcaa	transposase, ISlin1
LIC10927	-144	-132	cgatTTTGCATCTCAAAtctt	putative lipoprotein
LIC10953	-22	-10	atctTTTGAAACCCAAAttgg	hypothetical protein
LIC10968	-228	-216	tcaaTTTGTTGATCAAAgctg	SidA-regulated protein
LIC10996	-94	-82	agatTTTGAGTTTCAAActca	putative diguanylate phosphodiesterase
LIC11152	-46	-34	tttcTTTGAGATTCAAAaaaa	pseudouridylate synthase, 23S RNA-specific
LIC11196	-149	-137	aaaaTTTGTAATTCAAAattc	pyridoxine 5'-phosphate oxidase V related protein
maoC	-175	-163	catcTTTGTTACACAAAgagc	maoC dehydratase
LIC11448	-57	-45	cttcTTTGGCAATCAAAgcaa	transposase, ISlin1
LIC11471	-78	-66	cttcTTTGGCAATCAAAgcaa	transposase, ISlin1
recN	-198	-186	tcagTTTGGAAGACAAAagaa	DNA repair protein
fadD	-246	-234	tccgTTTGTAAAACAAAtaca	long-chain-fatty-acid CoA ligase
LIC11638	-159	-147	gaaaTTTGAAATCCAAAactg	hypothetical protein
dnaG	-22	-10	ccccTTTGTCCAACAAAaagg	DNA primase
recA	-232	-220	aattTTTGCTATACAAAtact	recombinase
yadG	-15	-3	gaagTTTGCGTTGCAAAttga	hypothetical protein
LIC11850	-262	-250	cactTTTGAATTTCAAAtcta	16S ribosomal RNA methyltransferase
metX	-258	-246	ttttTTTGTATTTCAAAggag	homoserine O-acetyltransferase
LIC11925	-129	-117	atatTTTGCGATTCAAAcaag	phage terminase
LIC12040	-62	-50	taaaTTTGACATTCAAAaatt	phnB-like
panD	-117	-105	atacTTTGAATACCAAAattc	aspartate alpha-decarboxylase
LIC12235	-208	-196	tttcTTTGTTTTACAAAgaac	histidine kinase response regulator hybrid protein

LIC12236	-218	-206	gttcTTTGTAAAACAAAgaaa	hypothetical protein
pdxJ	-22	-10	caatTTTGAAAGTCAAActca	pyridoxine 5'-phosphate synthase subunit
LIC12399	-41	-29	agagTTTGTAGATCAAAattc	hypothetical protein
LIC12587	-170	-158	aaagTTTGGGGAGCAAAcagc	putative lipoprotein
LIC12708	-260	-248	acagTTTGACAGGCAAAgaca	pertactin
LIC12793	-168	-156	atcgTTTGTGTTTCAAAaatc	biotin-(acetyl-CoA carboxylase) ligase
pyrB	-26	-14	tcaaTTTGATTGTCAAAaggg	aspartate carbamoyltransferase catalytic subunit
LIC12987	-202	-190	aaatTTTGATACGCAAAatct	enterotoxin A
sulP	-176	-164	agatTTTGTATCTCAAAaaga	sulfate permease
LIC12993	-88	-76	cggtTTTGTTTTTCAAAacag	high affinity receptor for IgE Fc
LIC13063	-78	-66	cttcTTTGGCGATCAAAgcaa	transposase, ISlin1
LIC13067	-78	-66	cttcTTTGGCGATCAAAgcaa	transposase, ISlin1
LIC13071	-96	-84	ttcaTTTGATTTTCAAAtccg	phosphatidylethanolamine-binding protein domain
LIC13101	-146	-134	atacTTTGGATTCCAAAgttt	alpha inegrin repeat-containing outer membrane protein
LIC13114	-82	-70	cttcTTTGGCGATCAAAgcaa	transposase, ISlin1
LIC13131	-264	-252	ttttTTTGCTCCTCAAAgagt	YceI
LIC13161	-78	-66	cttcTTTGGCGATCAAAgcaa	transposase, ISlin1
folK	-248	-236	cgatTTTGGAGTTCAAAaaga	amino-hydroxy-hydroxymethyldihydropteridine pyrophosphokinase
tas	-57	-45	ataaTTTGTTTTTCAAAtgtt	oxidoreductase
icdA	-167	-155	ttatTTTGAAATGCAAAatct	isocitrate dehydrogenase
LIC13254	-197	-185	gtaaTTTGTAATACAAAtcga	binding domain of leucine dehydrogenase
LIC13395	-71	-59	agatTTTGGACATCAAAgtta	OsmC-like protein
flgL	-240	-228	ggaaTTTGGAATTCAAAacga	flagellar hook-associated protein FlgL
LIC13461	-149	-137	ccttTTTGACGTACAAAcgga	putative lipoprotein
acpP	-194	-182	ccaaTTTGAAAAACAAAaacg	acyl carrier protein
LIC20198	-41	-29	atttTTTGAATCACAAAggct	hypothetical protein
yocS	-269	-257	tagaTTTGACGAACAAAtttt	bile acid Na^{+} symporter
